# Rational design of photosynthetic reaction center protein maquettes

**DOI:** 10.3389/fmolb.2022.997295

**Published:** 2022-09-21

**Authors:** Nathan M. Ennist, Steven E. Stayrook, P. Leslie Dutton, Christopher C. Moser

**Affiliations:** ^1^ Department of Biochemistry and Biophysics, University of Pennsylvania, Philadelphia, PA, United States; ^2^ Institute for Protein Design, University of Washington, Seattle, WA, United States; ^3^ Department of Biochemistry, University of Washington, Seattle, WA, United States; ^4^ Department of Pharmacology, Yale University School of Medicine, New Haven, CT, United States; ^5^ Yale Cancer Biology Institute, Yale University West Campus, West Haven, CT, United States

**Keywords:** *de novo* protein design, electron transfer, heme, porphyrin binding, protein structure, artificial photosynthesis, photochemical charge separation, redox chemistry

## Abstract

New technologies for efficient solar-to-fuel energy conversion will help facilitate a global shift from dependence on fossil fuels to renewable energy. Nature uses photosynthetic reaction centers to convert photon energy into a cascade of electron-transfer reactions that eventually produce chemical fuel. The design of new reaction centers *de novo* deepens our understanding of photosynthetic charge separation and may one day allow production of biofuels with higher thermodynamic efficiency than natural photosystems. Recently, we described the multi-step electron-transfer activity of a designed reaction center maquette protein (the RC maquette), which can assemble metal ions, tyrosine, a Zn tetrapyrrole, and heme into an electron-transport chain. Here, we detail our modular strategy for rational protein design and show that the intended RC maquette design agrees with crystal structures in various states of assembly. A flexible, dynamic apo-state collapses by design into a more ordered holo-state upon cofactor binding. Crystal structures illustrate the structural transitions upon binding of different cofactors. Spectroscopic assays demonstrate that the RC maquette binds various electron donors, pigments, and electron acceptors with high affinity. We close with a critique of the present RC maquette design and use electron-tunneling theory to envision a path toward a designed RC with a substantially higher thermodynamic efficiency than natural photosystems.

## Introduction

The advent of oxygenic photosynthesis *c*. 3 billion years ago has been called the “big bang of evolution” because it precipitated a new age of abundant life under an oxidizing atmosphere (Barber and Tran, 2013; Barber, 2016; Sanchez-Baracaldo and Cardona, 2020). Biology solves the solar energy storage problem by using photosynthetic reaction centers (RCs) to produce oxygen and hydrogen equivalents (fuel) from water splitting. RCs allow life to tap into the >10^8^ GW supply of solar power available at the surface of the Earth (World Energy Council, 2013). In contrast, energy storage remains a challenge for solar photovoltaics and other forms of renewable energy, making artificial photosynthesis an important objective for 21st century chemistry.

The RC executes a series of electron-transfer reactions to convert solar photons into chemical energy. The minimalist RC design is a chain of at least three cofactors, one of which is light-activatable, that are connected by electron tunneling through the insulating protein framework. A photoexcited pigment (*P*) in the middle of the RC reduces an electron acceptor (*A*) and oxidizes an electron donor (*D*), creating a charge-separated *D* ^+^ *PA* ^−^ state that can be used for redox chemistry such as water oxidation in Photosystem II (PSII). RCs maintain their near-unity quantum yields (Wraight and Clayton, 1974) by tuning the rates of electron tunneling to favor forward electron transfer while preventing electron-hole recombination. To estimate rates of thermodynamically downhill electron-tunneling reactions, the following empirical expression was developed from studies of natural RCs and other redox active proteins (Moser et al., 1992; Page et al., 1999; Moser et al., 2010):

$$\log_{10}\left(k_{ET}\right)=13-0.6\left(R-3.6\right)-3.1\frac{{\left(\Delta G+\lambda \right)}^{2}}{\lambda }$$ (1)

Equation 1

The rate constant *k*_ET_ is given in s^−1^, the edge-to-edge distance *R* between redox centers is in Å, and units of eV are used for both the reorganization energy λ and the driving force of the reaction Δ*G*. For uphill reactions (positive Δ*G*), the log rate is slower by Δ*G*/0.06 eV, maintaining a Boltzmann ratio between the downhill and uphill rates (Woodbury et al., 1986).

Several model systems have been developed to study RCs. Synthetic photochemical *DPA* triads reproduce the multi-step electron-transport activity of native photosystems (Megiatto et al., 2014; Imahori et al., 2001), and various proteins have been engineered for charge separation starting from native or *de novo* scaffolds (Conlan et al., 2009; Hingorani et al., 2014; Kalman et al., 1999; Farid et al., 2013; Soltau et al., 2015; Tebo et al., 2021). However, no previous *de novo*-designed protein has all the elements of a *DPA* electron-transport chain. A charge-separating RC maquette with substitutable *DPA* cofactors can serve as a useful testbed to examine the fundamental properties of RCs without the arduous task of isolating and stabilizing native photosystems.

Our long-term goal is to progressively develop a catalytically active *de novo* RC that self-assembles *in vivo* for enhanced biofuel production. Natural photosystems have high activities and efficiencies, but *de novo* RCs could be designed for improved suitability for commercial biofuel production. Natural photosynthesis stores solar energy in ATP and carbohydrates which need to be converted to other fuels before commercial use. An RC could be engineered to produce a fuel that requires less downstream processing. A new RC powered by near infrared or green light might complement the absorption cross-sections of native photosynthetic complexes such as PSI and PSII which have similar absorbance profiles and therefore compete for the same solar photons (Blankenship et al., 2011). Finally, the photoexcited P680 pigment of PSII has a low enough redox potential to reduce protons or carbon dioxide, meaning that it is possible to split water into an oxidant and a fuel in a single photosystem (Barber and Tran, 2013; Mersch et al., 2015). The one-photosystem model would shorten the extensive photosynthetic electron-transport chain (which loses free energy as electrons flow down an electrochemical potential gradient) and preclude the need for absorption of a second photon by PSI before fuel production.

Recent progress in computational protein design and structure prediction has been rapid and stands to transform our approach to *de novo* protein design in general (Jumper et al., 2021; Baek et al., 2021). The ever-increasing predictive power of new computational methods will continue to refine our understanding of the protein sequence-structure relationship. The role of human intuition will nevertheless remain significant in the design of electron-transfer proteins for some time to come. RC design is more complex than a protein folding problem because of other important considerations such as cofactor binding and specificity, electron-transfer rates, inter-cofactor distances, excited state energies and lifetimes, redox potentials, and reorganization energies. For some protein scaffolds including four-helix bundles, adherence to simple design rules is sufficient to achieve the intended tertiary structure. The choice of a robust, designable helical bundle fold makes it easier to address the many other factors necessary for efficient light-activated charge separation.

We use “maquettes,” scaled-down *de novo*-designed proteins with simple scaffolds, to help tease out design rules for various protein functions. Iterative experimentation and modification of maquette designs allow biochemists to develop their understanding of protein activity and subsequently develop more advanced functions (Lichtenstein et al., 2012; Ennist et al., 2017; Moser et al., 2016; Grayson and Anderson, 2018).

Progression from the first functionless four helix bundle maquettes in the 1980s to light-driven charge separation in the RC maquette illustrates the maquette approach in action. Early work showed that amino acid sequences with a binary pattern of hydrophobic (H) and polar (P) residues in a heptad repeat (i.e. HPPHPPH or HPPHHPP) could be engineered to fold into four-helix bundle proteins (Regan and DeGrado, 1988; Kamtekar et al., 1993). The addition of metal-ligating histidine residues to the hydrophobic core enabled binding of various metalloporphyrins, opening the door to cofactor-mediated electron transfer and light responsive chemistry (Robertson et al., 1994; Rau and Haehnel, 1998; Sharp et al., 1998). Assembly of both heme B and a Zn porphyrin into the same maquette enabled intra-protein light-driven electron transfer with a short-lived charge separated state (Farid et al., 2013). The four-helix bundle scaffold was also engineered to host a diiron binding site analogous to that of bacterioferritin, ribonucleotide reductase, and other natural oxidoreductases (Summa et al., 1999; Faiella et al., 2009; Torres Martin de Rosales et al., 2010; Lahr et al., 2005; Lombardi et al., 2019). The DeGrado group recently combined a di-metal center and a Zn porphyrin pigment binding pocket into the same scaffold (Pirro et al., 2020). These advances build upon one another to create maquettes with increasing complexity and functionality.

In this article, we detail the design process that produced a recently reported RC maquette (Ennist et al., 2022), investigate its cofactor binding activity, and analyze crystal structures in various stages of assembly. The RC maquette integrates a Co or Fe porphyrin electron acceptor (*A*-site), a Zn porphyrin pigment (*P*-site), and an electron donating di-metal center and tyrosine (*D*-site) for photoactive charge separation. In addition, we will present a vision for the next generation of RC maquettes: we aim to improve the quantum yield and thermodynamic efficiency of charge separation through the addition of low-potential electron acceptors and use of a photooxidative mechanism for polynuclear metal cluster assembly reminiscent of photoactivation in PSII (Bao and Burnap, 2016; Zhang et al., 2017; Vinyard et al., 2019; Cardona, 2016). Using Eq. 1 as a guide, the maquette approach permits the selection and arrangement of redox cofactors to optimize the preservation of chemical redox energy in the photosynthetic light-activated, charge-separated state.

## Materials and methods

### Protein expression and purification

A gene encoding the RC maquette was purchased from DNA2.0 in a pJexpress414 vector, and the DH maquette gene was purchased from GenScript in a pET-3a vector. Both genes included an N-terminal His_6_ tag followed by a TEV protease cleavage sequence. Invitrogen supplied mutagenesis primers, and mutant plasmids were PCR amplified with AccuPrime^™^ Pfx SuperMix (Invitrogen). Amino acid sequences are given in Supplementary Table S1.

Plasmids were transformed into BL21-(DE3) competent cells (New England Biolabs), and proteins were expressed and purified as previously described (Ennist et al., 2022). Where noted, an additional purification step of high performance liquid chromatography (HPLC) was executed on a Waters reverse-phase HPLC system using a C4 or C18 HPLC column (Grace Davison Discovery Sciences). HPLC-pure samples were lyophilized and resuspended in >6.5 M GdnHCl, refolded by dilution, and purified by size exclusion chromatography (SEC) with Superdex 75 prep grade gel filtration medium (GE Healthcare Life Sciences).

### Ultraviolet/visible spectroscopy and preparation of tetrapyrrole cofactors

Ultraviolet/visible absorbance spectroscopy (UV/vis) was carried out on a Varian Cary-50 spectrophotometer. Cofactor binding titrations were performed at room temperature using 1 ml samples of ∼1 μM RC maquette in quartz cuvettes. Protein concentrations were calculated by assuming that Trp and Tyr residues have extinction coefficients at 280 nm (ε_280nm_) of 5,500 M^−1^ cm^−1^ and 1,490 M^−1^ cm^−1^, respectively. Samples of Zn 5-phenyl 15-(*p*-carboxyphenyl) porphyrin (ZnPCP) and Zn Newkome porphyrin were provided by Tatiana Esipova from the research group of Sergei Vinogradov (Kodali et al., 2017). The Zn chlorins SE370 and SE375 were produced by the laboratory of Jonathan Lindsey following previously established methods (Aravindu et al., 2013; Kodali et al., 2017). Other tetrapyrroles were purchased from Frontier Scientific, Inc. Stock solutions of water-soluble cofactors were prepared in water, and insoluble tetrapyrroles were prepared in dimethyl sulfoxide stock solutions of 100–200 µM concentration. Heme B stock concentrations were measured by hemochrome assay (Berry and Trumpower, 1987). Stock concentrations of other tetrapyrrole cofactors were estimated by mass measurements.

For each titration, a cofactor stock solution was titrated into the protein solution in the cuvette. After each addition of cofactor, at least 10 min were allowed for equilibration before collecting a UV/vis spectrum. Cofactor was typically added in steps of 0.1 equivalents of cofactor per protein until after the binding capacity of the protein appeared to have been reached, at which point 0.2 equivalents of cofactor per protein were added at a time. Binding titrations continued until at total of at least three equivalents of cofactor per protein had been added. To determine dissociation constants, we used the following one-site binding equation:

$$A=c_{tot}{ɛ}_{free}+\left({ɛ}_{bound}-{ɛ}_{free}\right)\frac{K_{D}+P_{tot}+c_{tot}-\sqrt{{\left(K_{D}+P_{tot}+c_{tot}\right)}^{2}-4P_{tot}c_{tot}}}{2}+b$$ (2)

In Eq. 2, *A* represents the total absorbance at a given wavelength, *c*_tot_ is the total cofactor concentration, *P*_tot_ is the total protein concentration, *K*_D_ is the dissociation constant, ε_free_ is the extinction coefficient of unbound cofactor in solution, ε_bound_ is the extinction coefficient of protein-bound cofactor, and *b* is the absorbance of the sample when *c*_tot_ is zero.

Estimates of the amounts of heme B incorporated into proteins during overexpression were made by UV/vis measurements. The extinction coefficient of heme B at the Soret maximum (ε_415nm_) was found to be 115,000 M^−1^ cm^−1^ for hemes in bis-His binding sites in the RC maquette and DH maquette. The ε_280nm_ of heme B bound to a maquette is approximately 20,000 M^−1^ cm^−1^, allowing estimation of the ratio of heme to maquette protein after purification.

### Circular dichroism spectroscopy

An Aviv Model 410 instrument was used for circular dichroism (CD). Ultraviolet CD spectra were collected from 190 to 260 nm. The bandwidth used was 1 nm, and data points were measured every 1 nm with 5 s averaging time per data point. Samples were prepared in 0.1 cm path length quartz cuvettes. Each reported spectrum represents the average of three spectra measured in succession. Thermal denaturation experiments measured the CD signal at 222 nm for 5 s with a 2 nm bandwidth, and data were collected every 2°C with a heating rate of 10°C/min followed by a 4-min incubation time. The mean molar ellipticity per residue, θ_MMR_, is the measured ellipticity divided by 196 residues (the length of the RC maquette).

### Crystallography

We report two new crystal structures deposited in the PDB under accession codes 8D9O and 8D9P. The 8D9O protein stock solution was purified by HPLC to remove residual heme B leftover from expression in *E. coli*. Heme B was added to the 8D9P protein sample as described previously (Ennist et al., 2022). Protein stock solutions contained 20–40 mM NaCl and 10–20 mM piperazine-N,N′-bis(2-ethanesulfonic acid) (PIPES) buffer at pH 6.5. Crystals were grown in hanging drops in which 1 µl of well solution was added to 1 µl of protein stock solution. Supplementary Table S2 gives protein stock concentrations, well solutions, cofactors, and cryoprotectants used for the two crystals. Both X-ray diffraction data sets were obtained from single crystals grown at 4°C and cooled to 100 K for data collection. A Rigaku Micromax-007 HF rotating copper anode X-ray generator with VariMax HF optics and a Rigaku Saturn 944 HG CCD detector collected X-ray diffraction data. Phaser (McCoy et al., 2007) performed molecular replacement using the crystal structure with PDB ID: 5VJS as a search model (Ennist et al., 2022). Structure solution and refinement was carried out using the CCP4i (Winn et al., 2011) and PHENIX (Adams et al., 2010; Liebschner et al., 2019) software packages. Scaling of diffraction intensities was done in SCALA (Evans, 2006). Structures were refined using REFMAC5 (Vagin et al., 2004), phenix.refine (Afonine et al., 2012), and PDB_REDO (Joosten et al., 2014). RESOLVE performed density modification (Terwilliger, 2000), and Coot facilitated real space refinement (Emsley et al., 2010). Polder omit maps in Supplementary Figure S2 were generated in Phenix (Liebschner et al., 2017). Data collection and refinement statistics are given in Supplementary Table S3.

### Electron-tunneling simulations

Equation 1 is used to calculate the electron-tunneling rate for exergonic electron transfers. The reverse, endergonic reaction applies a rate penalty of one order of magnitude for every 0.06 eV of endergonic free energy. These rates are used to generate a set of differential equations solved in Wolfram Mathematica, Version 11.1.1 for electron transfers between cofactors in the tetrad and pentad kinetic schemes. The time zero boundary condition begins with the singlet excited pigment. Supplementary Table S5 provides the inter-cofactor edge-to-edge distances, excited state energies and decay lifetimes, reorganization energies, and redox midpoint potentials used in the calculations. Driving forces for electron transfer are estimated from the midpoint potentials given in Supplementary Table S5.

### Computational modeling of protein structure

**FIGURE 1:**
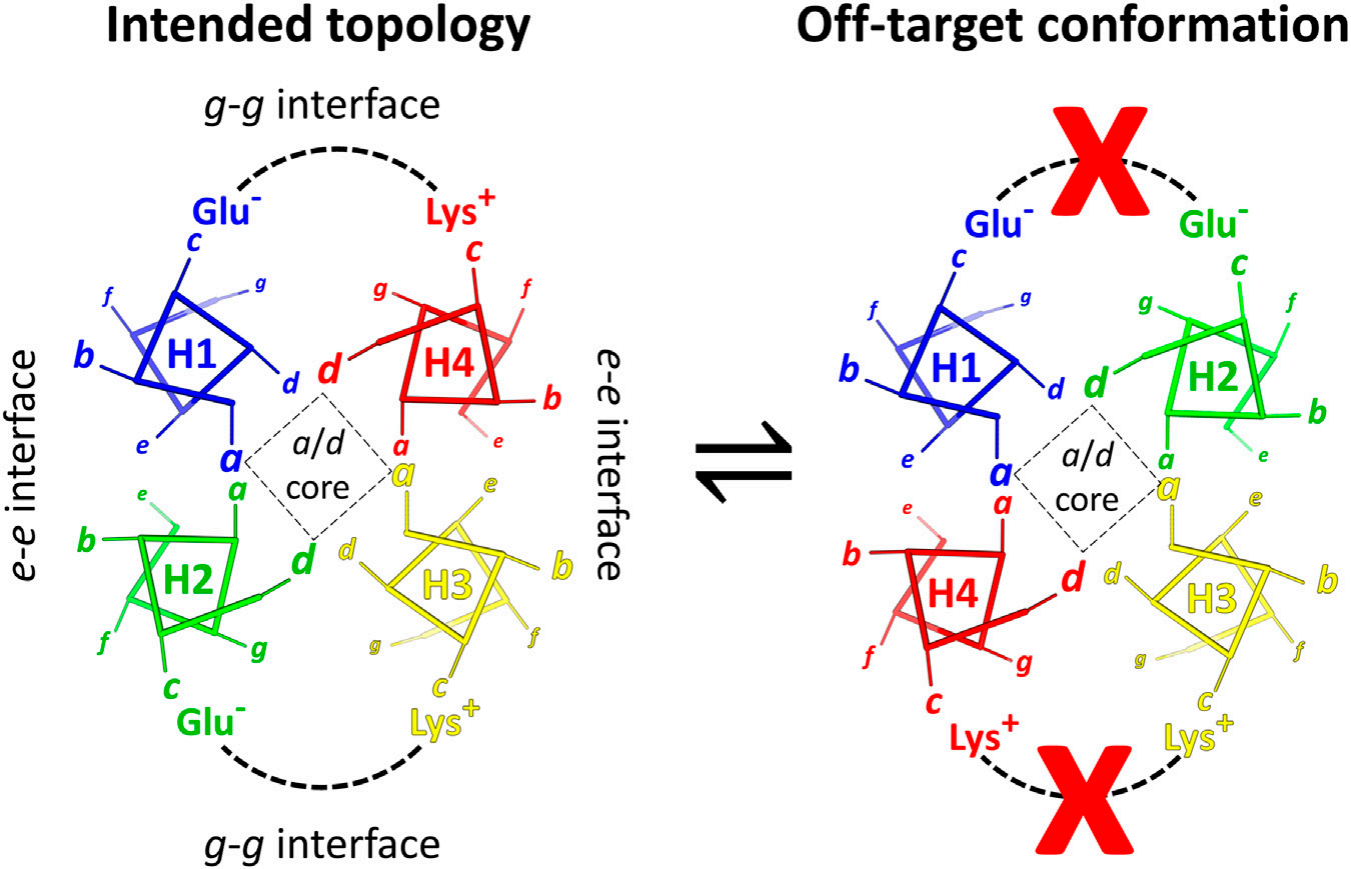
Helical wheel diagrams show how charge pairing interactions disfavor a misfolded state. Left: the correctly-folded antiparallel coiled coil structure has favorable Glu – Lys interactions. Right: helices H2 and H4 are transposed in the misfolded state, creating Glu – Glu and Lys – Lys charge clashes as indicated by the red Xs. For each helix, one example heptad is displayed. The distance of an amino acid from the viewer is represented by the size of its heptad letter designation. In helices, thick sticks connect C_α_ atoms, while thinner lines represent C_α_-C_β_ bonds.

Comparisons between protein structures were made by aligning the structures and calculating the root mean square deviations between aligned C_α_ atoms (C_α_ RMSD) using TM-align (Version 20190822) (Zhang and Skolnick, 2005). Structure prediction with RoseTTAFold was done using the server at https://robetta.bakerlab.org (RoseTTAFold option) (Baek et al., 2021). Structure prediction with AlphaFold2 (Jumper et al., 2021) was done in ColabFold without multiple sequence alignments (Mirdita et al., 2022). The impact of charge complementarity in the RC maquette as illustrated in Figure 1 was evaluated for two mutant designs containing six point mutations each. The first mutant switches the *c*-position charged amino acids in the porphyrin binding domain in helices 1 and 3: E8K, E15K, E22K, K109E, K116E, and K123E. The second mutant switches *c*-positions of helices 2 and 4: E80K, E87K, E94K, K177E, K184E, and K191E.

## Results

### Modular design of a reaction center maquette

We conceived the RC maquette as a four-helix coiled coil protein for two primary reasons. First, helical bundles are highly designable. Decades of studies have refined the design rules and shown how diverse amino acid sequences can adopt this fold (Regan and DeGrado, 1988; Harbury et al., 1993; Robertson et al., 1994; Kamtekar et al., 1993; Kamtekar and Hecht, 1995; Moser et al., 2016; Grigoryan and Degrado, 2011; Rojas et al., 1997; Gibney et al., 1999; Huang et al., 2014; Boyken et al., 2016; Walshaw and Woolfson, 2001; Kumar and Woolfson, 2021). Approximately 5% of known natural protein sequences contain coiled coils (Rackham et al., 2010), including some that serve as excellent models for the RC maquette. Bacterioferritin, for example, contains a diiron site with a nearby tyrosine amino acid (Conlan et al., 2009; Hingorani et al., 2014), and cytochromes (cyt.) *b* and *b*_6_ (from the cyt. *bc*_1_ and *b*_6_ *f* complexes, respectively) assemble two porphyrins within electron-transfer distance of one another (Malone et al., 2019). Secondly, a four-helix bundle is an ideal scaffold because it is conducive to the iterative redesign of an RC maquette. Inter-cofactor distances can be adjusted by translating binding sites in increments of approximately one helical turn along the bundle axis. Since cofactor binding sites are largely independent, modules can be added or removed. Simply lengthening the helices will extend the bundle and allow it to assemble more redox centers into a longer chain. Analogous manipulations in many other protein folds would be prohibitively difficult.

Several key principles of four-helix bundle design derived from previous studies contributed to the RC maquette design process. They include *1*) binary patterning in heptad repeats, *2*) a balance of high helix-propensity vs. beta-branching/aromatic residues, *3*) negative design using charge complementarity, *4*) shape complementarity in the hydrophobic core, and *5*) attention to the structural details of analogous native and designed proteins. We elaborate on these five points below.

#### Binary patterning

The RC maquette employs a binary pattern of hydrophobic and polar amino acids in heptad repeats throughout its helices to help stabilize a coiled coil fold (Kamtekar et al., 1993). In contrast to straight α-helices that have about 3.6 amino acids per helical turn, canonical coiled coils wrap α-helices around each other like a rope to create a left-handed supercoil with almost exactly 3.5 residues per turn (Grigoryan and Degrado, 2011; Crick, 1953b; Crick, 1953a). As a result, there are precisely two turns per heptad of amino acids, and every seventh residue finds itself in a nearly equivalent local environment. Following convention, we designate the amino acid positions in the RC maquette heptads *a* through *g*, such that *a-* and *d*-positions are the most buried in the hydrophobic core. With exceptions for cofactor ligation, the RC maquette uses hydrophobic residues in the buried *a*- and *d*-positions and polar residues in exposed *b*-, *c*-, and *f*-positions. Hydrophobicity of interfacial *e*- and *g*-positions vary along the helices according to the needs of nearby cofactor binding sites.

#### Amino acid composition

To promote the folding of a uniquely-structured, stable coiled coil, the RC maquette hosts a balance of different types of amino acids in buried *a*-, *d*-, *e*-, and *g*-positions. Amino acids with high α-helical propensities, such as Ala, Met, and Leu, have a strong preference for α-helical phi/psi angles (Pace and Scholtz, 1998), but an excess of small or flexible residues often confers a poorly ordered and non-native-like hydrophobic core. Inflexible aromatic and β-branching residues, including Phe, Tyr, Ile, Thr, and Val, help to limit the conformational freedom of the core and constrain it to a single unique structure (Betz and DeGrado, 1996; Raleigh and Degrado, 1992; Gibney et al., 1999; Kamtekar and Hecht, 1995). Helical segments of the RC maquette include 44 Ala and Leu residues (22% of the protein) to promote helix formation and 20 aromatic and β-branching residues (10% of the protein, not including His) to minimize conformational freedom.

#### Negative design

Negative design using charge complementarity can stabilize the correctly folded state relative to off-target conformations. In Figure 1, a diagram of the RC maquette on the left shows helices 1–4 arranged counterclockwise when viewed from the electron accepting end, but an unintended clockwise topology shown on the right could also form a canonical antiparallel coiled coil. This off-target conformation would have a similar overall structure with favorable core packing and knob-into-hole interactions, but the metal binding site would be distorted. One strategy to favor the intended topology is to create an energy gap between the two alternative states using strategic placement of charged amino acids (Cochran et al., 2005; Summa et al., 2002; Marsh and DeGrado, 2002). In the RC maquette, surface-exposed *c*-positions in the pigment/electron acceptor region were chosen to be charged Glu or Lys such that they would form stabilizing salt bridges in the designed counterclockwise topology and destabilizing charge clashes in the clockwise state (Figure 1). Uncharged Gln residues were selected for most *b*-, *e*-, and *f*-positions in the porphyrin domain to avoid electrostatic interference.

The impact of charge pairing was evaluated by structure prediction using AlphaFold2 in CoLabFold (Jumper et al., 2021; Mirdita et al., 2022). AlphaFold2 accurately predicts the experimentally-validated counterclockwise arrangement of helices in the RC maquette with high confidence (consistent with Figure 1, left). However, structure prediction becomes more ambiguous when six substitution mutations rearrange the distribution of *c*-position Glu and Lys residues of the porphyrin-binding domain to favor the reversed (clockwise) helix threading. Structures of the mutants are predicted with lower confidence, and AlphaFold2 produces some models with the reversed topology. (*See* methods for details). This illustrates the utility of inter-helical electrostatic interactions in determining tertiary structure.

#### Hydrophobic core packing

**FIGURE 2:**
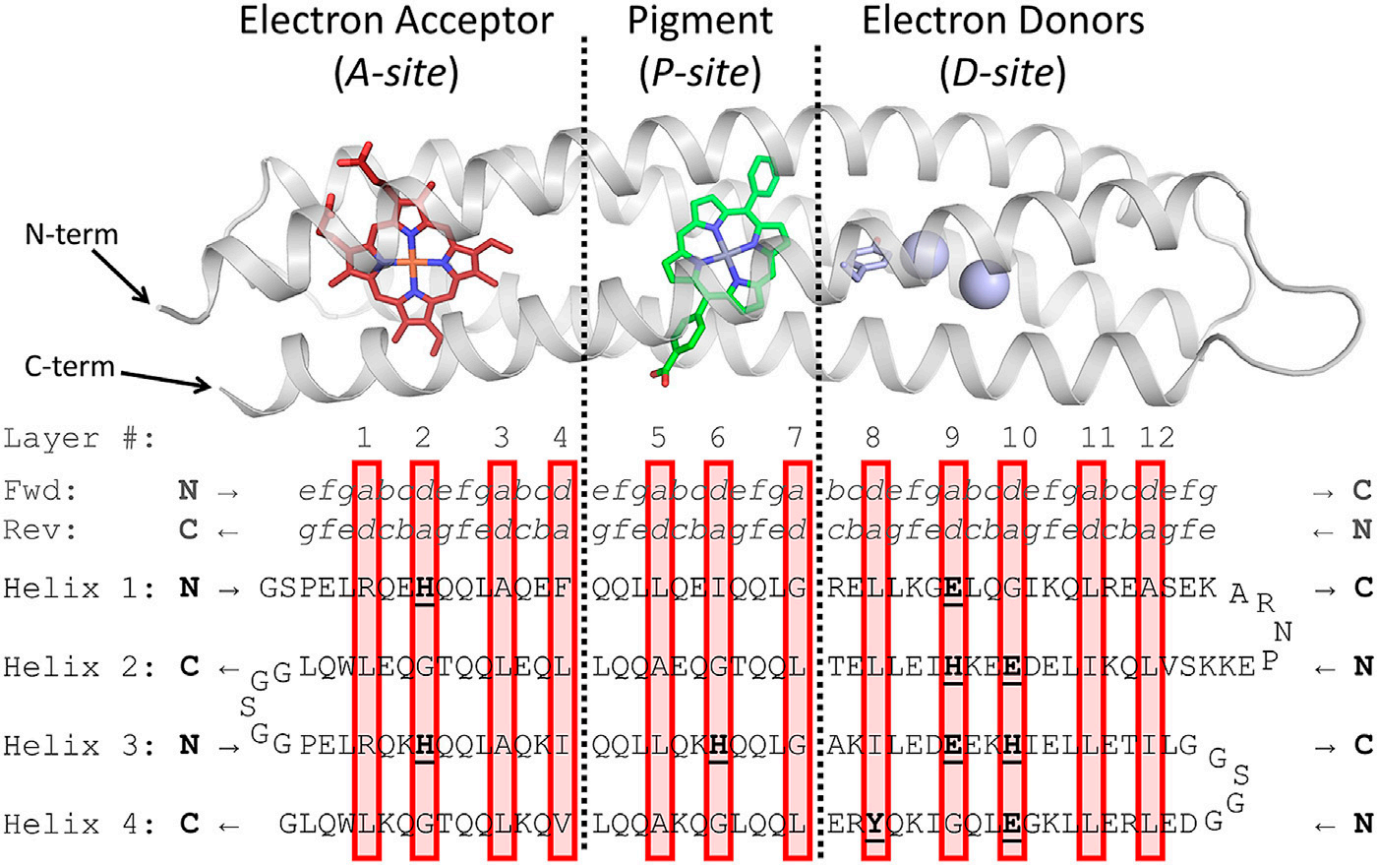
Design of RC maquette amino acid sequence. Above, holo-state model of RC maquette design is shown with electron acceptor in red, pigment in green, and electron donors in light blue. Below, amino acid sequence of RC maquette is written with each helix on a new line. Helices 2 and 4 are written backwards from C to N-terminus (bold black letters at ends of each helix indicate N- or C-terminus of that helix). (Full forward sequence is given in Supplementary Table S1). Amino acids in bold and underlined are directly involved in cofactor assembly. Heptad positions *a* through *g* are given above the protein sequence for forward and reverse directions. Red boxes enclose layers of the hydrophobic core (numbered above). Each layer includes two *a*- and two *d*-position residues.

The primary driving force for folding of most proteins is the hydrophobic effect, but complementary packing of hydrophobic residues that are far apart in sequence space can be difficult to predict. Early maquettes solved the problem by using Leu residues almost exclusively in *a*- and *d*-positions (Regan and DeGrado, 1988; Robertson et al., 1994); the flexible side chains with uniform size ensured that the folded state would have a hydrophobic core without destabilizing cavities. However, Leu flexibility also allowed multiple conformational states to exist simultaneously, which complicates characterization efforts (Raleigh and Degrado, 1992; Gibney et al., 1999). Today, computational methods such as AlphaFold (Jumper et al., 2021), RoseTTAFold (Baek et al., 2021), and Rosetta (Leman et al., 2020) can meet the challenge of core packing, but an intuitive, logical approach to sequence selection affords the designer a firmer grasp of the role of each amino acid. A deeper understanding of the protein structure and the reasoning behind the sequence facilitates iterative redesign. One approach is to consider the hydrophobic core of the helical bundle as a stack of layers, where each of the four helices contributes one amino acid to each layer (Munson et al., 1994; Betz and DeGrado, 1996; Betz et al., 1997; Liu et al., 2007; Gernert et al., 1995; Chino et al., 2015; Willis et al., 2000; Summa et al., 1999; Walshaw and Woolfson, 2001; Glover et al., 2014). In the antiparallel RC maquette coiled coil, each layer of the core consists of two *a*- and two *d*-position residues (Figure 1). The layers, indicated by red boxes in Figure 2, are designed to contain a roughly consistent cumulative volume of amino acid side chains and cofactors to avoid introducing cavities or bulges that would destabilize the core. For instance, layer *4* in Figure 2 has large Phe, Ile, Val, and Leu residues, but layer *2* offsets the bulk of its porphyrin electron acceptor ligated by two *d*-position His residues with small *a*-position Gly residues. Not all coiled coils have unambiguously identifiable layers, but in many cases layers help the protein designer recognize and understand patterns of core packing.

#### Using natural proteins as templates

Distinct *a*/*d* layers are observable in native proteins including cyt. *b* and *b*_6_, as illustrated in Supplementary Figure S1 (Hunte et al., 2000; Malone et al., 2019). Despite being membrane proteins, these two-heme cytochromes serve as useful templates for the design of the pigment/acceptor module of the RC maquette. Their edge-to-edge distance between hemes is close to the optimal *P*-to-*A* distance for long-lived charge separation in the RC maquette (Ennist et al., 2022). Sequence alignments reveal that the *a*/*d* layer between the hemes of cyt. *b* and *b*_6_ (corresponding to layer *4* in the RC maquette; *see* Figure 2) typically consists of bulky hydrophobic residues that serve to widen the helical bundle so that it can accept the heme cofactors (Rau and Haehnel, 1998; Esposti et al., 1993). Adjacent to this central layer are two heme-contacting layers (corresponding to layers *3* and *5* in Figure 2). They usually include smaller or more flexible amino acids such as Ala and Leu which make space for the heme. The heme-ligating layers (corresponding to layers *2* and *6* in the RC maquette) use *d*-position His to coordinate the heme iron and small *a*-position “notch glycines” that offset the bulk of the hemes and His residues (Saribas et al., 1997). While Gly residues are generally more destabilizing in water soluble proteins than in membrane helices, studies have shown that protein maquettes can be designed to support multiple helical glycines and remain stable (Torres Martin de Rosales et al., 2010; Faiella et al., 2009; Ghirlanda et al., 2004; Rau and Haehnel, 1998). The RC maquette includes a total of 10 α-helical glycine residues that were selected to optimize core packing and cofactor binding. The sequence identity between the RC maquette and natural membrane *b*-type cytochromes is negligible, but attention to the packing of layers in the hydrophobic core helps give the RC maquette a similar overall structure in the porphyrin-binding module.

Antiparallel four-helix bundles that bind porphyrins are common in nature (Hunte et al., 2000; Malone et al., 2019; Mathews et al., 1979; Weber et al., 1980; Yankovskaya et al., 2003; Dautant et al., 1998), and experimental structures of *de novo*-designed versions have been published recently (Polizzi et al., 2017; Pirro et al., 2020; Mann et al., 2021; Hutchins et al., 2020). One common trend in all of the examples cited here is that the plane of the porphyrin ring cuts through the *e*-*e* interface of the helical bundle, causing the *e*-*e* interface to expand while the *g*-*g* interface can remain narrow. This preference may be the result of structural differences between the two interfaces; in *g*-positions, the C_α_-to-C_β_ bond points toward the hydrophobic core, whereas *e*-position C_α_-to-C_β_ bonds tend to direct the side chains away from the core, enabling *e*-position residues to more easily avoid clashing with the porphyrin cofactor. Both *e*- and *g*-positions are interfacial and can be either polar or nonpolar in four-helix bundles. We chose mostly polar Gln for *e*-positions in the porphyrin-binding module, and *g*-positions are mostly Leu except for three *g*-position Thr that make second shell hydrogen bonds to His ligands.

An electron donating metal site and tyrosine are found in native carboxylate-bridged diiron four-helix bundle proteins such as bacterioferritin. The DeGrado and Lombardi groups reproduced this distinctive fold in a series of *de novo*-designed “Due Ferri” maquettes and engineered a variety of functions including ferroxidase and phenol oxidase activity (Summa et al., 1999; Faiella et al., 2009; Torres Martin de Rosales et al., 2010; Lahr et al., 2005; Pirro et al., 2020; Chino et al., 2015; Lombardi et al., 2019). Given the success of this line of research, Due Ferri 3 (DF3) was selected as the basis for sequence design in the electron donor module of the RC maquette (Faiella et al., 2009; Torres Martin de Rosales et al., 2010). DF3 has a higher affinity for various metal ions than previous Due Ferri proteins owing to its four active site Gly residues that impart greater flexibility to the metal-coordinating amino acids (Torres Martin de Rosales et al., 2010). To avoid potential side reactions in the RC maquette, Tyr and Trp amino acids were replaced by redox-inactive residues, with a single Tyr kept as an intermediate electron donor between the metal center and pigment binding site. To avoid complications from nonspecific cofactor ligation, a DF3 loop containing a His residue was replaced with a loop from DF2t (Lahr et al., 2005). A flexible Gly-rich linker was added to incorporate the homodimeric Due Ferri sequence into the single chain RC maquette (*see* Figure 2).

To fuse the electron donating di-metal/Tyr portion of the RC maquette to the pigment/electron acceptor domain, we joined the corresponding helices together to make an extended 7-nm long antiparallel four-helix bundle. Helix-helix interface widths differ between the two domains. The di-metal/Tyr region has a narrow *e*-*e* interface that must be widened in the pigment/acceptor domain in order to allow binding of the tetrapyrroles. To facilitate variations in the coiled coil Crick parameters across the RC maquette, each helix has a buried Gly residue at the junction between the two domains to increase backbone flexibility. These four Gly residues also create space for binding of the Zn porphyrin pigment (similar to notch glycines in cyt. *b* and *b*_6_) and prevent hydrophobic collapse of the pigment binding site in the apo-state. Independently, the DeGrado group joined two maquettes to make a slightly shorter helical bundle containing one Zn porphyrin and a di-metal site for a different purpose; the Zn porphyrin acted as an allosteric regulator of ferroxidase and oxidase activity in the diiron site (Pirro et al., 2020). In the RC maquette, the addition of an electron accepting porphyrin enables the redox centers to function collectively as a light-driven electron-transport chain.

Figure 2 provides the overall design architecture of the RC maquette and the amino acid sequence. In all, the RC maquette is 196 residues long with a molecular weight of 22.5 kDa and a theoretical isoelectric point of 5.5. The results presented below give support to the design strategy outlined in the present section by providing experimental evidence of cofactor binding activity, a demonstration of modularity in the design, and high resolution X-ray crystal structures.

### Cofactor binding in the RC maquette

#### Binding of heme and other electron acceptors

**FIGURE 3:**
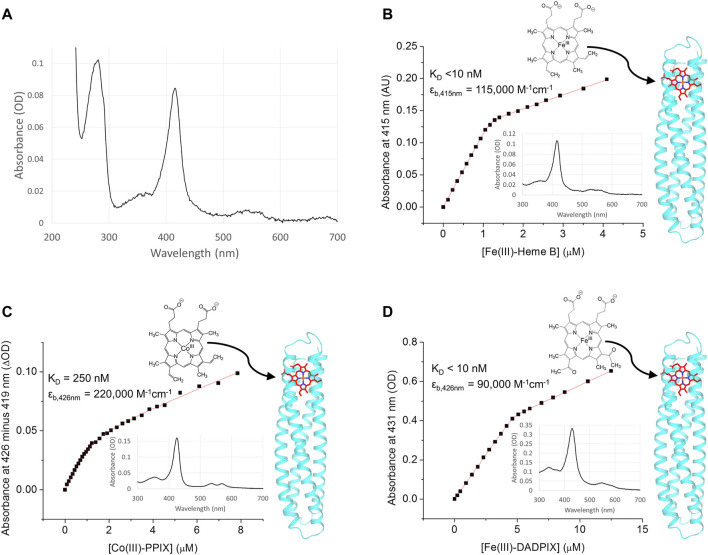
RC maquette electron acceptor binding. **(A)** Spectrum of purified RC maquette without addition of exogenous heme reveals that ∼10% of protein retained heme from expression in *E. coli* after protein purification. In panels **(B**–**D)**, absorbance is plotted against cofactor concentration and fit to a one-site binding equation (Eq. 2). The structure of each acceptor is shown, and arrows indicate the likely binding location in the RC maquette on the right. Inset are absorbance spectra after the addition of 0.8 molar equivalents of cofactor. **(B)** Binding of Fe(III)-heme B to 1.2 µM RC maquette in 15 mM NaCl and 10 mM sodium phosphate buffer at pH 7.0. **(C)** Binding of Co(III)-PPIX to 1.1 µM RC maquette in 50 mM NaCl and 10 mM MOPS buffer at pH 7.0. **(D)** Binding of Fe(III)-DADPIX to 4.4 µM RC maquette in 50 mM NaCl and 20 mM sodium phosphate buffer at pH 7.0.

Expression of the RC maquette in *E. coli* produced red cell pellets suggestive of high-affinity *in vivo* heme binding. The RC maquette retained its red color through Ni-NTA and SEC column chromatography. After purification, ∼0.1 equivalents of ferric heme B per mole of RC maquette remained bound, as estimated from UV/vis spectra (Figure 3A). HPLC removed heme from RC maquette samples prior to binding assays.

The RC maquette binds iron tetrapyrroles and cobalt tetrapyrroles through the use of two axial histidine ligands (His9 and His110) and hydrophobic contacts flexible enough to accommodate different alternative cofactors. Binding titrations monitored by UV/vis spectroscopy take advantage of the pronounced changes in metalloporphyrin spectra upon His ligation to quantify RC maquette affinities for various electron acceptors. Figure 3B plots the absorbance of a solution of 1.2 μM RC maquette at 415 nm against ferric heme B concentration. Fitting to Eq. 2 yields a dissociation constant (K_D_) of <10 nM and shows that heme binds with the intended stoichiometry of 1.0 heme per maquette. The related iron porphyrin Fe(III) 2,4-diacetyl deuteroporphyrin IX (Fe(III)-DADPIX) binds with a comparable affinity and stoichiometry. Replacement of the ferric ion of heme B with a cobaltic ion gives Co(III) protoporphyrin IX (CoPPIX), an electron acceptor that has been shown to be capable of hydrogen evolution upon reduction (Sommer et al., 2016). Like heme B, CoPPIX accepts two axial His ligands. A spectroscopic titration shows that the RC maquette binds 1.0 CoPPIX per protein with a K_D_ of 250 nM (Figure 3C). This is about 25 times weaker than the heme B affinity of the RC maquette, but comparable to the CoPPIX affinity of the native hemoprotein cyt. *b*_562_, which has a K_D_ in the range of 8.9 µM (Sommer et al., 2016) to 45 nM (Alcala-Torano et al., 2021). Despite the presence of His residues at other sites in the RC maquette, none of the electron acceptors tested showed signs of binding outside the designed acceptor binding site. The roles of His9 and His110 in heme coordination were verified by X-ray crystallography (*vide infra*).

#### Pigment binding

**FIGURE 4:**
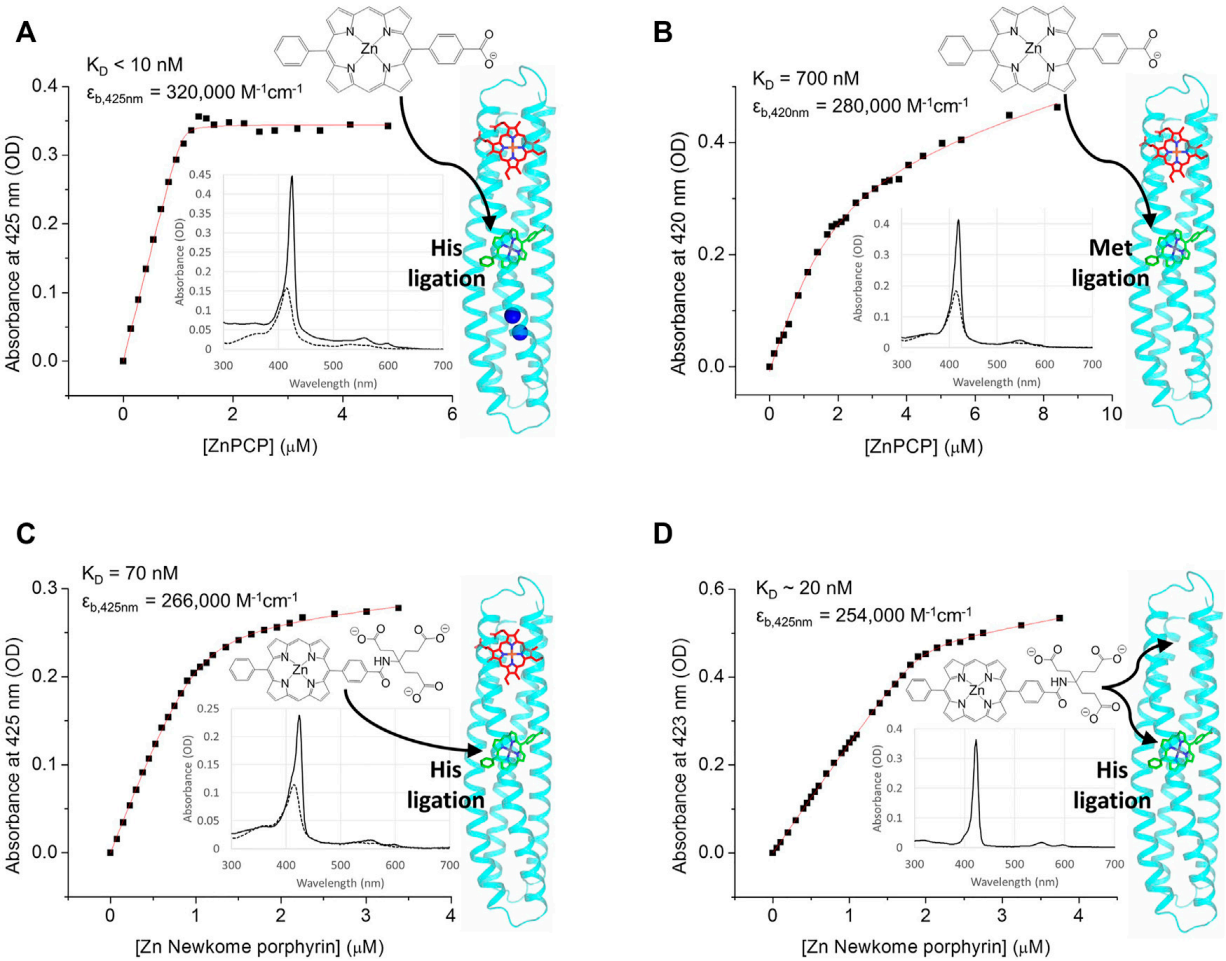
RC maquette pigment binding assayed by UV/vis absorbance titrations. For each plot, absorbance at the pigment Soret maximum (with heme absorbance subtracted out) is plotted against pigment concentration and fit to a one-site binding equation (Eq. 2). The structure of the pigment is shown, and arrows indicate its likely binding location in the RC maquette on the right. Inset are spectra before pigment binding (dotted lines, with heme) and after the addition of 0.8 molar equivalents of pigment (solid lines). In panels **(A**–**C)**, a protein solution was prepared with 1.0 molar equivalent of heme B bound before titrating in the pigment; in each case 1.0 pigment molecule binds per protein-heme complex. **(A)** Binding of ZnPCP to 1.2 µM RC maquette-heme with 1.5 mM ZnCl_2_. Conditions: 15 mM NaCl and 10 mM PIPES buffer at pH 6.5. **(B)** Binding of ZnPCP to 1.4 µM His124Met mutant RC maquette-heme. Conditions: 50 mM NaCl and 10 mM MOPS buffer at pH 7.5. **(C)** Binding of Zn Newkome porphyrin to 1.0 µM RC maquette-heme. Conditions: 15 mM NaCl and 10 mM sodium phosphate buffer at pH 7.0. **(D)** Binding of Zn Newkome porphyrin to 0.51 µM RC maquette that does not contain heme **(B)**. In the absence of heme, the stoichiometry is at least three Zn Newkome porphyrins per protein, suggesting a structural distortion in the maquette when pigment is added without an electron acceptor cofactor. Conditions: 15 mM NaCl and 10 mM sodium phosphate buffer at pH 7.0.

The RC maquette is designed to bind a light-activatable tetrapyrrole pigment in its central pigment binding site, the *P*-site, through the use of hydrophobic contacts and ligation by His124. To test for pigment binding, RC maquette samples were prepared with heme B already bound, and Zn tetrapyrrole binding was monitored spectroscopically as the pigments were titrated into the protein-heme solutions (Figures 4A–C). Zn 5-phenyl,15-(*p*-carboxyphenyl) porphyrin (ZnPCP) exhibits a pronounced red shift in its Soret maximum from 409 nm in buffer to 422 nm upon His ligation with a further shift to 425 nm in the presence of metal dications including Mn(II), Fe(II), and Zn(II) (Ennist et al., 2022). Fitting to Eq. 2 reveals that ZnPCP binds with the expected stoichiometry of 1.0 and a K_D_ of <10 nM in the presence of Zn(II).

Substitution of methionine (Met) for His124 weakens the ZnPCP affinity to 700 nM with a Soret maximum at 420 nm (Figure 4B). The alteration of ZnPCP absorbance and binding kinetics in the His124Met mutant provides evidence that residue 124 coordinates the Zn ion. Met ligation of Zn chlorin e_6_ has been confirmed in a modified bacterioferritin (Conlan et al., 2009; Hingorani et al., 2014), but to our knowledge, Met ligation of Zn tetrapyrroles has not been tested in maquettes previously. The more hydrophobic Met ligand might be expected to raise the pigment midpoint potential relative to His-ligated pigments as it does for heme B in other maquettes (Moser et al., 2016), offering a possible strategy to fine tune driving forces for electron-transfer reactions. Transient spectroscopy results reported previously indicate an increased quantum yield of charge separation in a Met124 RC maquette variant relative to His124 constructs (Ennist et al., 2022).

Several different Zn tetrapyrrole pigments were tested for binding to the RC maquette (with His124) with heme B bound to the acceptor site. Two Zn diphenyl porphyrins and two Zn diphenyl chlorins bind stoichiometrically with K_D_s in the nM range, and several other Zn porphyrins display weak (μM-range K_D_) or no detectable affinity. Comparison of the strong- and weak-binding pigments in Supplementary Table S4 reveals a pattern of structural constraints that can be used to predict affinities. As observed previously in other maquettes, high-affinity tetrapyrrole cofactors tend to have amphiphilic character that allows partitioning into the hydrophobic binding site of the protein without loss of aqueous solubility (Solomon et al., 2014; Kodali et al., 2017). However, in the pigment binding site of the RC maquette, the shape of the cavity confers additional selectivity. Consideration of spectroscopic and crystallographic data suggests that Zn meso-tetraphenyl porphyrins are too bulky to fit into the pigment site. Similar to previous porphyrin-binding maquettes with hydrophobic binding sites (Fry et al., 2010; Cochran et al., 2005; Bender et al., 2007), Zn protoporphyrin IX and Zn chlorin e_6_ have weaker affinities likely due to carboxylate groups close to the tetrapyrrole ring that resist burial. The four high-affinity Zn tetrapyrroles have a shape that better conforms to the contours of the binding cavity, and their polar groups are more distant from the central Zn ion and do not need to become buried upon binding.

Owing to the structural similarity between Zn tetrapyrrole pigments and the Fe and Co tetrapyrrole electron acceptors, some of the Zn tetrapyrroles tested were able to bind to both the pigment site and acceptor site (Figure 4D). For this reason, it was necessary to add the electron acceptor first in order to block pigment access to the acceptor site. While acceptor site promiscuity enables testing of different cofactors with varying redox potentials for electron-transfer studies (Ennist et al., 2022), future *in vivo* self-assembly of a working RC maquette will require greater binding selectivity in the acceptor site to avoid improper assembly of the electron-transport chain.

#### Metal binding

Carboxylate-bridged di-metal maquettes are typically capable of binding a variety of transition metals (Torres Martin de Rosales et al., 2010). For the purposes of an RC maquette, the most interesting metals are redox-active cations such as Mn(II), Fe(II), and Co(II). Binding of these dications was confirmed spectroscopically in the presence and absence of porphyrin cofactors as described in (Ennist et al., 2022). To summarize, Mn(II) binding was shown to increase the thermostability of the RC maquette, as measured by circular dichroism. Co(II) exhibited an absorbance maximum at 545 nm with an extinction coefficient of 92 M^−1^cm^−1^ when bound to the RC maquette, consistent with 5-coordinate Co(II) binding as expected (Bertini and Luchinat, 1984; Torres Martin de Rosales et al., 2010). The RC maquette was shown to have ferroxidase activity typical of designed and native diiron proteins (Yang et al., 2000; Pasternak et al., 2001). When ZnPCP and heme B are present, Mn(II) binding elicited a 3-nm shift in the Soret band that is reversible upon addition of EDTA. These results illustrated the intended metal binding behavior in the RC maquette (Ennist et al., 2022).

### Porphyrin-only DH maquette provides evidence of design modularity

The four-helix coiled coil scaffold facilitates modular design; amino acid selection tailored for specific cofactor binding at one site is largely independent of other sites. The RC maquette is composed of a cyt. *b*/*b*_6_-like pigment/electron acceptor (*P*/*A*) module and a bacterioferritin-like electron donor (*D*) module. The *D* module is based on the Due Ferri family of homodimeric maquettes, which function independently of a *P*/*A* module (Faiella et al., 2009; Torres Martin de Rosales et al., 2010; Lombardi et al., 2019; Chino et al., 2015). In contrast, the *P*/*A* module was designed from scratch with guidance from studies of natural and designed porphyrin-binding proteins as described above.

**FIGURE 5:**
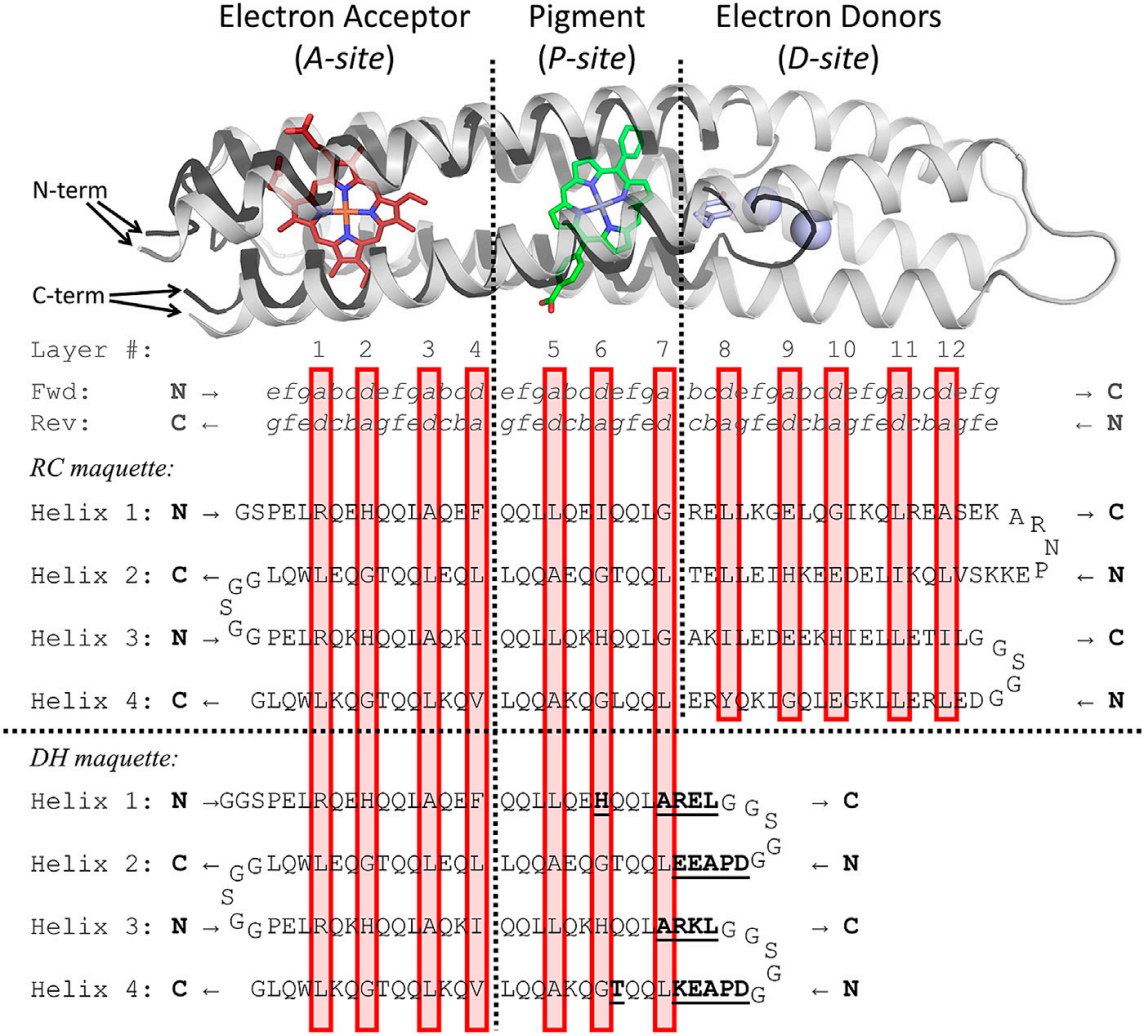
Sequence and structure comparison of RC and DH maquettes. Above, an RC maquette model based on crystal structures in white helices is overlaid with the RoseTTAFold (model 1) predicted structure of the DH maquette in black helices. The RoseTTAFold prediction has a high confidence score of 0.85. (AlphaFold predicted models are similar). The sequence diagrams below align the RC and DH maquette sequences. Differences between the maquettes in helical segments are indicated in bold and underlined in the DH maquette sequence. As in Figure 2, coiled coil heptad positions *a* through *g* are denoted in the top two rows, and *a*/*d* layers of the hydrophobic core are indicated by red boxes. The amino acid sequences have each helix written on a new line with helices 2 and 4 written in reverse from C-terminus to N-terminus as indicated by arrows and bold N or C at the ends of each helix. (Full forward sequences are given in Supplementary Table S1).

**FIGURE 6:**
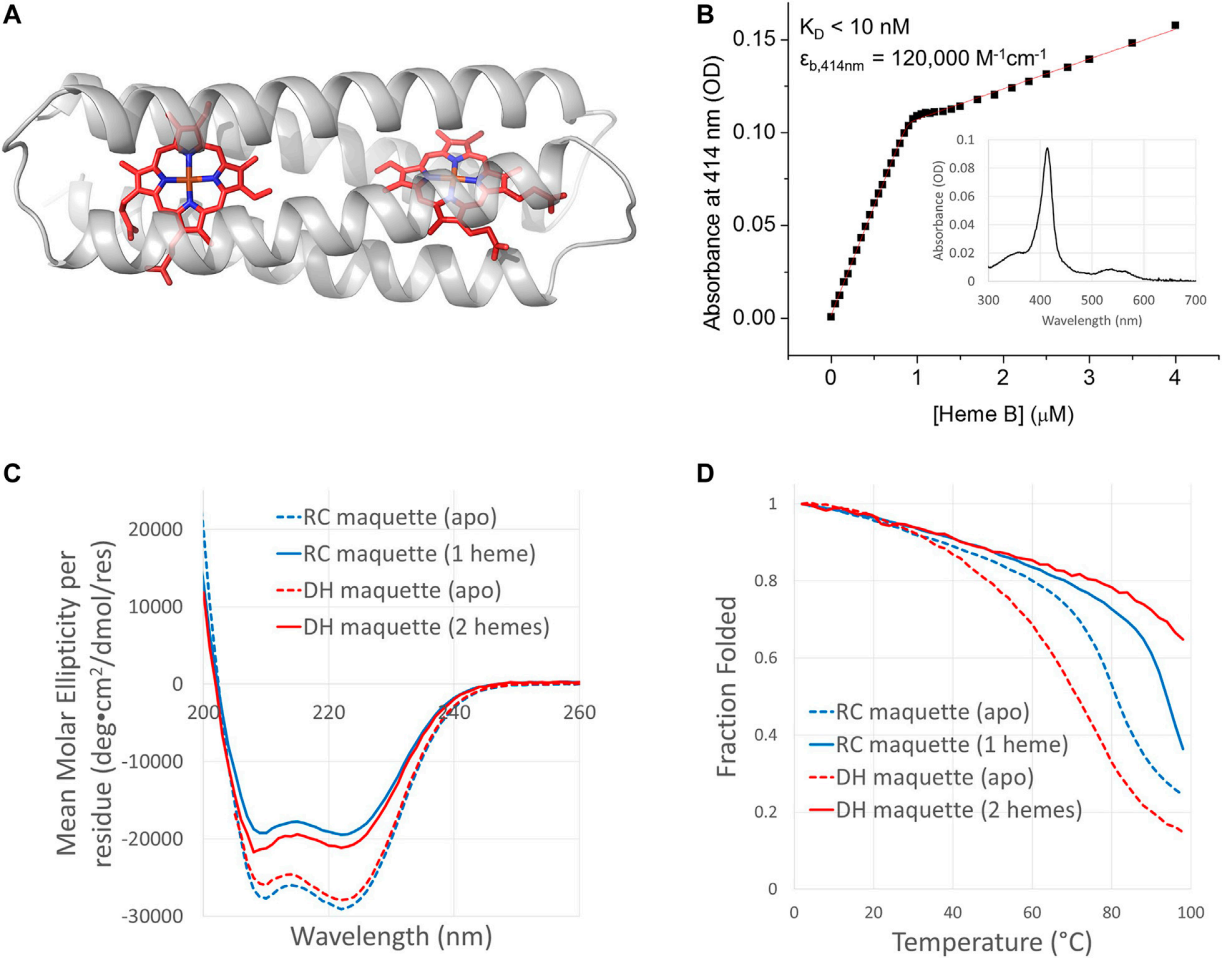
Design and function of the Di-Heme (DH) maquette. **(A)** DH maquette design model is based on crystal structure of RC maquette. **(B)** UV/vis binding titration of ferric heme B into 0.5 µM DH maquette was monitored at the Soret maximum at 414 nm. Inset: Spectrum at 0.8 molar equivalents of heme B per DH maquette protein. Conditions: 50 mM NaCl, 10 mM MOPS buffer, pH 7.5. CD spectra in panel **(C)** and thermal melts in panel **(D)** were measured in samples containing 15 mM NaCl and 10 mM NaH_2_PO_4_ at pH 7. Heme binding significantly increases the melting temperature but lowers the ellipticity at 222 nm. Protein concentrations were as follows: 16.8 µM apo-RC maquette (blue dashed curves), 20.3 µM RC maquette-heme B (blue solid curves), 31.8 µM apo-DH maquette (red dashed curves), and 18.3 µM DH with two hemes B (red solid curves).

To examine the *P*/*A* module separate from the *D* module and to illustrate the validity of our modular approach to design, we developed the Di-Heme maquette (DH) as an independent version of the *P*/*A* module of the RC maquette (Figures 5, 6). The DH maquette conserves 96% of the sequence of the *P*/*A* module but inserts loops and N-terminal caps to account for the absence of the *D* module. In addition, a His and second-shell Thr were substituted into the pigment binding site to convert it into a second heme site. While sequence-based structure prediction using AlphaFold (Jumper et al., 2021) or RoseTTAFold (Baek et al., 2021) was not available when the DH maquette was designed, retrospective structure prediction of DH from its sequence produces apo-state models in good agreement with the *P*/*A* module of RC maquette crystal structures (<2.0 Å C_α_ RMSD).

IPTG-induced overexpression of the DH maquette in *E. coli* produced red cell pellets similar to the RC maquette. After purification by immobilized metal affinity chromatography, cleavage of the N-terminal His-tag using TEV protease, and SEC, UV/vis spectra showed that the DH maquette contained ∼0.25 molar equivalents of heme B (or ∼0.13 equivalents per binding site) without addition of exogenous heme. Heme was removed prior to heme binding titrations using HPLC. Binding titrations using UV/vis spectroscopy revealed a stoichiometry of two hemes binding to each DH protein with a dissociation constant of <10 nM (Figure 6).

CD measurements show the DH maquette to be highly α-helical and thermostable. Its T_M_ is 71°C in the apo-state and greater than boiling when two hemes B are bound. Addition of heme B decreases the measured ellipticity at 222 nm at 25°C from –27,900 deg cm^2^ dmol^−1^ res^−1^ in the apo-state to –21,100 deg cm^2^ dmol^−1^ res^−1^ when two hemes per protein are present, a 24% change (Figure 6C). This loss of ellipticity upon heme binding is similar to that observed in the RC maquette, but it stands in contrast to many other designed porphyrin-binding four-helix bundle proteins in which cofactor binding intensifies the ellipticity at 222 nm, presumably by promoting folding (Ghirlanda et al., 2004; Rau and Haehnel, 1998; Polizzi et al., 2017; Bender et al., 2007; Hutchins et al., 2020). One exception is the highly stable 4 PA maquette, in which a small decrease in ellipticity at 222 nm upon porphyrin binding was attributed to a porphyrin transition (McAllister et al., 2008). Given that crystal structures of the RC maquette show comparable α-helix content with and without heme (*vide infra*), the decreased ellipticity at 222 nm upon heme binding may represent a porphyrin transition or a subtle rearrangement of the tertiary structure rather than loss of secondary structure.

The DH maquette exhibits many properties similar to the *P*/*A* module of the RC maquette. Both have high α-helix content, increase their melting temperatures to above boiling upon cofactor binding, have comparable heme-induced changes in their CD spectra, bind heme in *E. coli* and retain it through the purification process, and bind porphyrins stoichiometrically with low-nM dissociation constants or better. While the high-resolution structure of the DH maquette has not yet been solved experimentally, machine learning methods predict accurate folding. These results indicate that the *P*/*A* module of the RC maquette is capable of functioning as an independent protein that does not rely on the *D* module.

### Crystal structures of RC maquette in various stages of cofactor assembly

The RC maquette was intended to fold as a canonical left-handed coiled coil with antiparallel α-helices. Since the maquette was designed with minimal use of computational methods, we did not produce an atomistic design model *a priori* to which we can compare crystal structures. To evaluate the accuracy of our design methods, we fitted the RC maquette crystal structure to Crick parameters using the Coiled-Coil Crick Parameterization (CCCP) tool (Grigoryan and Degrado, 2011; Crick, 1953a). The amino acid backbone in the helical region of the holo-state RC maquette crystal structure (PDB ID: 5VJS) deviates from an idealized four-helix coiled coil by 0.96 Å C_α_ RMSD. The crystal structure averages 3.494 residues per α-helical turn, very close to the intended 3.5 residues per turn. Other crystal structures of the RC maquette have similar Crick parameters. The agreement between the overall fold and an idealized coiled coil reflects our adherence to well-established design rules and the natural predisposition of helical bundles toward forming left-handed coiled coils.

#### Structure and flexibility of electron acceptor binding site

**FIGURE 7:**
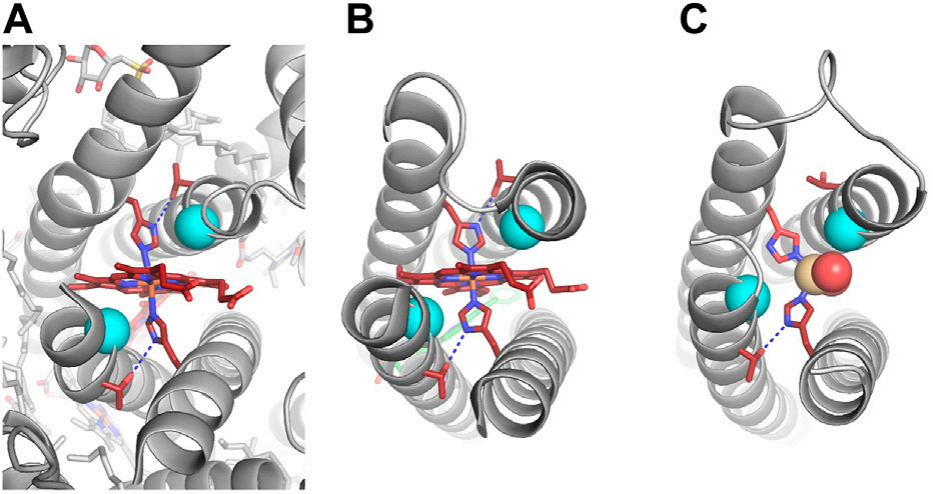
Experimental structures with and without heme. Heme B, ligating His residues, and second-shell Thr residues are shown in dark red. Notch Gly residues that make van der Waals contact with the porphyrin ring have C_α_ atoms shown in cyan. Hydrogen bonds are indicated by dashed blue lines. **(A)** Cryo-electron microscopy structure of spinach cytochrome *b*_6_ *f* (PDB ID: 6RQF) (Malone et al., 2019). **(B)** X-ray crystal structure of RC maquette with heme B and a Zn porphyrin (PDB ID: 5VJS) (Ennist et al., 2022). **(C)** X-ray crystal structure of RC maquette without heme B at 1.78 Å resolution (PDB ID: 8D9O). Cd(II) is shown as a tan sphere coordinated by the two histidines and two water molecules (red spheres). In the heme-free structure, the *A*-site is narrower, and the imidazole ring of His110 is flipped, breaking the hydrogen bond to Thr91.

The *P*/*A* module of the RC maquette with heme B and ZnPCP bound (PDB ID: 5VJS) matches the cyt. *b*_6_ subunit of cyt. *b*_6_ *f* (PDB ID: 6RQF) to within 2.6 Å C_α_ RMSD, despite having only 14% sequence identity in the 125-residue aligned region (Malone et al., 2019) (Figure 7). Residues His9 and His110 in the RC maquette coordinate the Fe(III) of the heme ligand with their N_ε_ atoms; the His N_δ_ atoms are backed up by hydrogen bonds to second-shell Thr residues as designed. The notch Gly residues make the intended van der Waals contacts with the porphyrin rings, and *a*/*d* layers of the hydrophobic core in the *P*/*A* module resemble those of cyt. *b* and *b*_6_ as intended.

**FIGURE 8:**
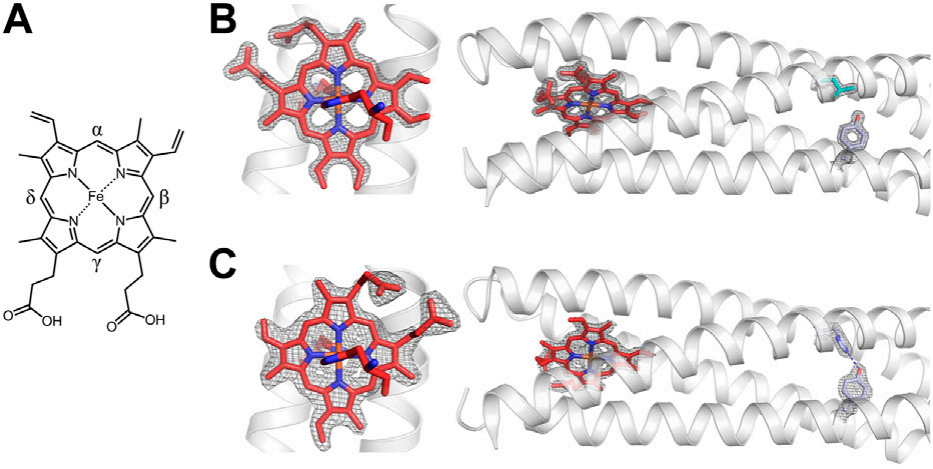
A Leu71His mutation distant from the *A*-site triggers a 90° rotation of heme B. **(A)** Chemical structure of heme B. **(B)** The 1.45 Å resolution crystal structure with heme B and Leu71 (PDB ID: 5VJT). Left: close-up of *A*-site is shown with 2.0 σ contouring of (F_obs_, φ_calc_) map around heme B. Right: larger context of same structure shows heme B, Leu71, and Tyr168 with 2.0 σ contouring. **(C)** The 2.08 Å resolution crystal structure with heme B and a His71-Tyr168 hydrogen bond (PDB ID: 5VJU). Left: close-up of *A*-site in Leu71His mutant is shown with 1.0 σ contouring of (F_obs_, φ_calc_) map around heme B. Right: larger context of same structure shows heme B and His71-Tyr168 hydrogen bond (indicated by blue dashed line) at 1.5 σ contouring.

RC maquette crystal structures reveal a highly accurate design, but they also provide evidence of conformational flexibility in the electron acceptor site. Heme B has a pseudo-C_2_ symmetry axis passing through the α and γ meso positions, but the symmetry of the molecule is broken by substituent vinyl groups (Figure 8A). Similar to some native heme proteins, the RC maquette does not strongly discriminate between the two possible 180° rotations about the α-γ meso axis (Mortuza and Whitford, 1997; Vallone et al., 2004). Even at 1.45 Å resolution (PDB ID: 5VJT), the positions of the vinyl groups are ambiguous (*see* Figure 8B).

Further evidence of conformational flexibility in the heme site is found in an RC maquette crystal structure with a Leu71His mutation (PDB ID: 5VJU). Relative to Leu71 structures, the mutant exhibits a 90° rotation of the heme about an axis passing through the heme iron and His N_ε_ atoms (Figures 8B,C). Despite the rotation, protein-cofactor contacts as well as inter-cofactor distances are unchanged. The cause of the change in heme conformation is unclear. The Leu71His mutation may trigger a subtle conformational shift that is translated more than 2 nm from the *D* module to the *A*-site. However, the most obvious conformational difference in the *A*-site of the mutant is a change in crystal contacts involving the heme propionate groups, suggesting that the two heme binding orientations are similar in energy in solution but that different crystal packing arrangements favor one state over the other.

A complete understanding of cofactor binding requires consideration of the apo-state structure and its transition to the holo-state. While the RC maquette crystallized more readily with heme bound, one heme-free structure was solved (PDB ID: 8D9O), which showed that the maquette retains its α-helical structure in the absence of heme (Figure 7C). The heme-bound and heme-free structures align with a C_α_ RMSD of 1.2 Å when inter-helical loops are not included in the alignment. The heme-free structure was obtained using a crystallization condition that included a large excess of CdCl_2_. Several Cd(II) ions are resolved in the structure, one of which effectively takes the place of the heme by coordinating the two *A*-site His residues as well as two water molecules. An additional water molecule in the *A*-site is hydrogen bonded to Gln111, and the side chains of Trp, Gln, and Leu residues adopt rotamers that would clash with the heme cofactor were it present. Measurement of the superhelical radius near the *A*-site using CCCP (Grigoryan and Degrado, 2011) indicates that the bundle widens to 7.8 Å radius in the heme-bound RC maquette (PDB ID: 5VJT) compared to 7.3 Å when Cd(II) occupies the *A*-site (PDB ID: 8D9O), a 7% change. Only one of the two His-Thr pairs in the *A*-site forms a hydrogen bond in the heme-free structure, whereas both hydrogen bonds form when heme is present. These structural differences suggest that the cofactor binding site is only partially pre-organized prior to heme binding.

Given that the *a*/*d* core of the RC maquette near the *A*-site includes two Gly, two His, and two Arg residues, all of which are likely to be strongly destabilizing in the apo-state, perhaps it is unsurprising that crystallization attempts without a stabilizing *A*-site ligand were unsuccessful. In a true apo-state where the *A*-site His residues do not coordinate a metal of any kind, it is likely that the *A*-site is more disordered. The decreased thermostability of the apo-protein (Figure 6) supports this conclusion.

While pre-organization of cofactor binding sites has been shown to aid binding specificity in some designed proteins (Ruckthong et al., 2016; Mills et al., 2013), pre-organization is not a universal requirement for high-affinity cofactor binding. Many native heme proteins employ a “partial fold option” for heme binding in which a partly-structured apo-protein with a disordered heme binding region collapses into a fully native-like structure only upon cofactor binding (Falzone et al., 1996; Landfried et al., 2007). This cofactor assembly strategy balances the apo-state stability needed to minimize the entropic cost of binding with the flexibility required for insertion of a bulky, hydrophobic porphyrin cofactor. Without the use of lyases to aid cofactor attachment as in the assembly of many biliproteins (Schluchter et al., 2010; Mancini et al., 2018), excessive stability might impede cofactor binding by requiring the disruption of strong protein-protein interactions to allow ligand entry into the binding pocket. Indeed, there is little pre-organization of heme sites in the apo-forms of myoglobin (Lin et al., 1994), cyt. *b*_5_ (Falzone et al., 1996), cyt. *b*_562_ (Feng et al., 1994), and cyt. P450_cam_ (Pfeil et al., 1993), all of which transition from a partially folded apo-state to a more structured, native-like holo-state. In the RC maquette, the high heme affinity and mostly folded but flexible apo-state indicate that the partial fold option has been applied successfully in a *de novo*-designed heme-binding protein.

#### Structure of pigment binding site

**FIGURE 9:**
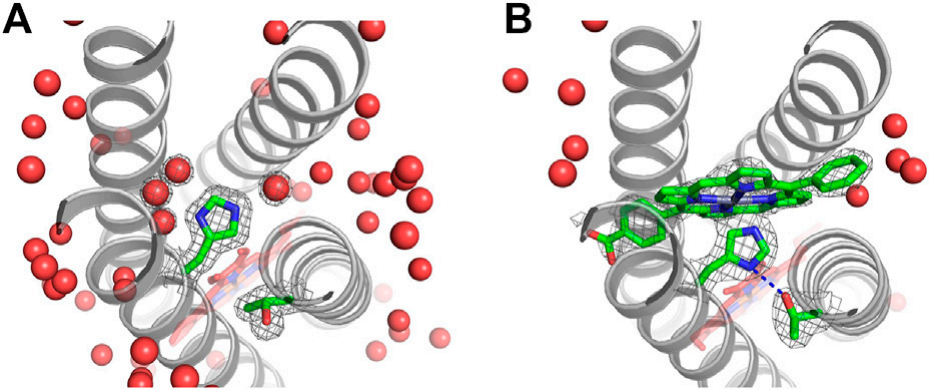
Structure of *P*-site with and without pigment bound. The (F_obs_, φ_calc_) electron density is shown at 1.0 σ contouring for His124, Thr77, and the pigment or water molecules in the *P*-site. **(A)** The 1.45 Å resolution crystal structure without a pigment bound includes four water molecules in the *P*-site (PDB ID: 5VJT). **(B)** A 2.0 Å resolution crystal structure with pigment ZnPCP at 70% occupancy reveals that upon pigment binding, the *P*-site widens slightly and water molecules are removed from the binding site (PDB ID: 5VJS). A blue dashed line indicates a hydrogen bond between Thr77 and His124 with 2.7 Å between heavy atoms.

The RC maquette *A*-site lends itself to the partial fold option as a strategy for cofactor binding because it is close to the end of the bundle where the helices tend to be more dynamic and can more easily widen to accept a bulky cofactor. In contrast, the *P*-site is located in the more rigid center of the protein. In the 2.0 Å resolution RC maquette crystal structure with ZnPCP (PDB ID: 5VJS), omit maps confirm the presence of the pigment in the *P*-site, and Multi-wavelength Anomalous Dispersion (MAD) phasing positively identifies the Zn(II) ion in the ZnPCP cofactor (Ennist et al., 2022). Four *P*-site Gly residues are meant to create space for the Zn porphyrin pigment, increase backbone flexibility, and prevent a premature hydrophobic collapse of the binding cleft that could obstruct cofactor binding. Without a Zn porphyrin pigment (PDB IDs: 5VJT, 5VJU, 8D9O, and 8D9P), four structured water molecules occupy the *P*-site; as intended, the binding cavity does not collapse in the apo-state (*see* Figure 9). In all RC maquette crystal structures, temperature factors of the *P*-site Gly residues are comparable to other residues in the vicinity of the site, suggesting that the Gly are not significantly mobile. However, upon binding of ZnPCP (PDB ID: 5VJS), the superhelical radius within one α-helical turn of the *P*-site expands by 4% (0.3 Å) as measured by CCCP (Grigoryan and Degrado, 2011), demonstrating moderate flexibility in the helical backbone.

The electron density for the ZnPCP pigment is not as well-defined as that for the heme B in the same crystal structure. This may be due to sub-stoichiometric occupancy of the ZnPCP (the pigment is assigned a 70% occupancy in the deposited structure). Alternatively, the ZnPCP may be more mobile than the heme because of the shape of the binding pocket or the fact that it has only one axial His ligand (His124) instead of two. Most natural proteins that use His imidazole side chains to bind metallotetrapyrroles such as chlorophylls and hemes use the His N_ε_ atom to coordinate the metal, probably because of steric limitations. However, there are rare examples of His N_δ_ ligation of hemes and chlorophylls in nature (Iverson et al., 1998; Umena et al., 2011; Jordan et al., 2001) and in earlier maquettes (Choma et al., 1994; Zhuang et al., 2004). Heme binding in the RC maquette *A*-site employs His N_ε_ atoms for heme iron ligation as designed (Figure 7). In the *P*-site, the electron density for the His124 ligand to ZnPCP suggests either a strained conformation or a mixture of states, such as ZnPCP-bound and unbound (PDB ID: 5VJS). The crystal structure is modeled with N_ε_ coordination of the ZnPCP in agreement with the design, but the geometry of ligation is not ideal. N_δ_ ligation is a poorer fit to the electron density, but remains a possibility. As modeled in the crystal structure, His124 positions its N_δ_ atom 2.7 Å from the hydroxyl oxygen of Thr77, suggesting a strong hydrogen bond when ZnPCP is bound. In contrast, crystal structures without ZnPCP clearly show that Thr77 is in a different rotameric state, and instead forms a hydrogen bond with the backbone carbonyl group of Thr73.

The process of Zn porphyrin pigment binding to the RC maquette *P*-site includes removal of four water molecules from the binding cavity, a 4% expansion of the superhelical radius around the *P*-site, and likely formation of a His-Thr hydrogen bond. The high ZnPCP affinity suggests that these modest conformational changes do not significantly impede pigment binding.

#### Structure of electron donor site

Crystal structures of the RC maquette show excellent agreement with the structure of bacterioferritin. The RC maquette *D* module with Cd(II) differs from Cd(II)-bacterioferritin by 1.25 Å C_α_ RMSD over a 96-residue aligned region (PDB IDs: 8D9O and 4CVS, respectively) (Hingorani et al., 2014). Both Cd(II) ions in the *D*-site have octahedral geometry. His67 and His138 coordinate the metal ions with their N_δ_ atoms and use their N_ε_ atoms to make hydrogen bonds with second-shell Asp residues as designed. Two axial bidentate Glu residues (Glu34 and Glu161) and two bridging Glu (Glu64 and Glu135) ligate the Cd(II) ions as shown in Supplementary Figure S2D. A bridging water or hydroxide ion coordinates both Cd(II) ions. The Tyr168 hydroxyl group makes a hydrogen bond with Glu34, and in the Leu71His mutant (PDB ID: 5VJU), Tyr168 also hydrogen bonds His71 as designed (Ennist et al., 2022).

A 1.90 Å-resolution Mn(II)-containing structure was obtained with heme B as the electron acceptor and no pigment (PDB ID: 8D9P). In this structure, the coordination geometry for the two Mn(II) ions matches that of the Cd(II)-containing structures, but with 5.0 Å between the Mn(II) ions. In contrast, the Cd(II) structures with and without heme B have the metals 3.6 Å apart (Supplementary Figures S2C,D). Despite this difference, crystallographic and spectroscopic data for RC maquette binding of Cd(II), Mn(II), and Co(II) support the conclusion that five amino acid ligands coordinate each metal ion.

While the Cd(II) and Mn(II) structures (grown at pH 4.6–5.9) are consistent with the design, crystal structures with Zn(II) show a different coordination geometry. All RC maquette crystals grew under acidic conditions. Given that protons are known to compete with metal binding in Due Ferri proteins (Pasternak et al., 2001), it is possible that acid affects the *D*-site in some structures. Crystals grown with Zn(II) at pH 4.4–4.5 (PDB IDs: 5VJS and 5VJT) have only one tetrahedral Zn(II) ion in the intended di-metal site (Ennist et al., 2022). The metal site furthest from the *P*-site is vacant, but a second tetrahedral Zn(II) ion is present on the outer surface of the RC maquette coordinated by the His138 N_ε_ atom and the two Asp residues that were intended as second-shell ligands.

Comparison of DF3, which has four active site Gly residues, with other Due Ferri maquettes that lack helical Gly residues showed that a flexible binding pocket can enhance affinity for various metals (Torres Martin de Rosales et al., 2010). However, some studies have argued that high stability and pre-organization in a designed metal site can improve selectivity to avoid off-target binding geometries (Ruckthong et al., 2016; Mills et al., 2013). In the RC maquette, metals bind with high affinity, but the four Gly residues in the *D*-site may be partly responsible for the observed conformational heterogeneity in different crystal structures. This suggests a tradeoff wherein a highly pre-organized binding site may strictly preserve the intended structure at the cost of binding affinity, and a flexible metal binding site may have high affinity but risk distortion of the structure.

### Critique of RC maquette and strategy to improve performance

The achievement of long-lived charge separation in the RC maquette represents an important step toward the *de novo* design of proteins for solar-to-fuel energy conversion (Ennist et al., 2022). However, several significant modifications need to be made before designed RCs become useful for renewable energy production. Future designs must avoid the use of triplet excited states, increase the thermodynamic efficiency and quantum yield of charge separation, and catalyze redox reactions for fuel production.

**FIGURE 10:**
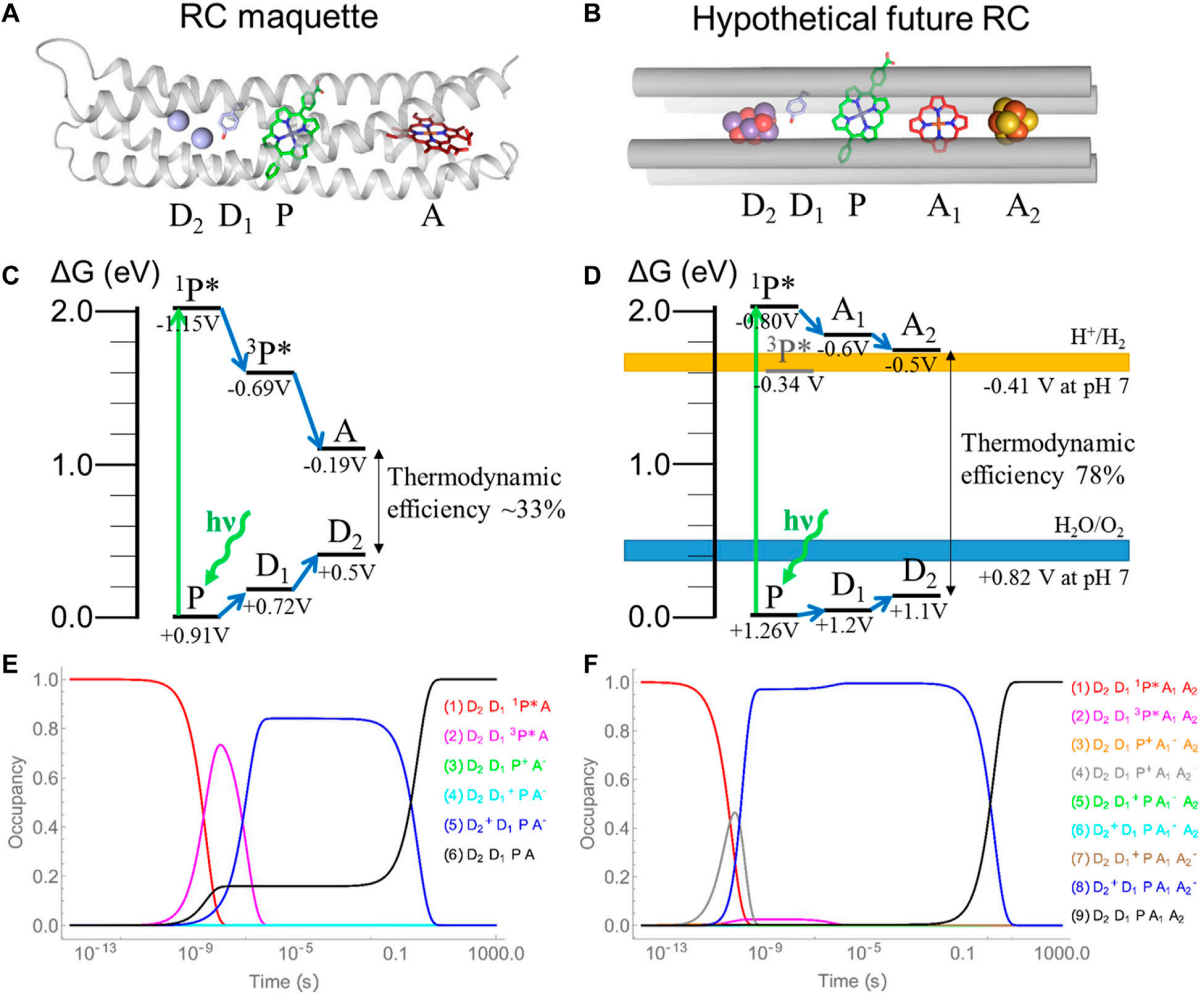
Comparison of RC maquette tetrad and hypothetical pentad design. **(A)** A model of the RC maquette tetrad with iron ions as the electron donor D_2_, a tyrosinate side chain as donor D_1_, Zn-5-(carboxyphenyl)-15-phenyl-porphyrin (ZnPCP) as the pigment P, and heme B as the electron acceptor A. **(B)** A diagram of a proposed pentad using a Mn_4_Ca cluster (similar to the OEC in PSII) as D_2_, tyrosine as D_1_, ZnPCP as P, a tetrapyrrole as A_1_, and an iron sulfur cluster as A_2_. **(C)** Energy level diagram of the RC maquette tetrad depicted in panel **(A)**. **(D)** Energy level diagram of the hypothetical pentad design depicted in panel **(B)**. **(E)** Predicted time evolution of the RC maquette tetrad after initial photoexcitation to the ^1^P* singlet state. **(F)** Predicted time evolution of the hypothetical pentad RC after photoexcitation to the ^1^P* singlet state. Potentials at which water oxidation and proton reduction occur are indicated by blue and orange bands, respectively. Calculations in panels **(E**,**F)** are based on Eq. 1 and do not consider the possible effects of changes in spatial distribution of electron density in the triplet state or spin rephasing.

The RC maquette exploits a Zn tetrapyrrole pigment with a relatively high-yield photoexcited triplet state. Promotion from the ground state to the singlet excited state is followed by intersystem crossing to the triplet excited state on a nanosecond timescale. The triplet state is a useful tool in the development of the RC maquette, because its millisecond-scale lifetime enables slow electron transfer over a long distance, allowing for easier characterization (Figures 10A,C,E). However, triplet states present many challenges, including susceptibility to oxidative damage by singlet oxygen and energy losses due to intersystem crossing. It has often been noted that electron density can be more spatially confined in triplet states than in singlet states (Richert et al., 2017), which will tend to slow initial charge separation. In addition, once the electron is transferred to heme, spin rephasing to a singlet charge separated state may lead to faster charge recombination. Indeed, transient absorption measurements of the RC maquette (Ennist et al., 2022) show that charge separation takes place even slower and with lower yield than the calculated electron-transfer behavior in Figure 10E, which does not take into account the shape of the triplet state electron cloud or spin rephasing effects. Additional inefficiencies in the RC maquette result from large potential differences between redox centers; the RC maquette tetrad charge separated state has a free energy difference of ∼0.69 eV between the oxidized Fe(III) donor and reduced ferrous heme B acceptor (Figure 10C), a ∼33% thermodynamic yield from the excited singlet state energy of 2.06 eV. By comparison, the thermodynamic efficiency of PSII is ∼44–56% (Vinyard et al., 2013).

Electron-tunneling calculations using Eq. 1 suggest that a much higher efficiency is attainable in a designed pentad. With ideal placement of electron-transfer cofactors and finely tuned redox potentials, the proposed pentad design in Figures 10B,D,F is predicted to have a 78% thermodynamic efficiency, a 99.5% quantum yield at 1 ms after excitation, and a charge separated state lifetime of 1.9 s. Its midpoint potentials for the tyrosine donor D_1_, metal cluster donor D_2_, and the pigment P/P^•+^ redox couple, are set to the same potentials as those in PSII (Vinyard et al., 2013). Lifetimes of intersystem crossing, internal conversion, and fluorescence are approximated as values typical of Zn porphyrins (Magdaong et al., 2020). The hypothetical pentad RC avoids the pigment triplet excited state by using a relatively short P-to-A_1_ distance of 5.7 Å to facilitate rapid electron transfer from the singlet state that outcompetes intersystem crossing. A secondary electron acceptor can prolong the lifetime of charge separation (Moser et al., 2017). In Figure 10B,D,F, we conceive A_1_ as a tetrapyrrole with a midpoint potential of −0.6 V and A_2_ as an iron-sulfur cluster with a midpoint of −0.5 V vs. SHE. The high thermodynamic yield gives the charge separated state enough energy for both water oxidation and hydrogen evolution in the same RC. A summary of parameters used for calculating electron-tunneling rates and efficiencies is given in Supplementary Table S5.

Certainly there are many challenges in the development of a pentad RC such as that proposed in Figure 10. Foremost among them is the photoassembly of an oxygen evolving cluster (OEC). The Mn_4_CaO_5_ OEC of PSII is assembled by successive cycles of metal ion binding and photooxidation (Bao and Burnap, 2016). In higher oxidation states, metals are more likely to form metal oxide clusters, so it may be possible to use an RC maquette to emulate the process of OEC photoassembly. Other major hurdles include tuning midpoint potentials, engineering catalytic sites, insulating the reducing and oxidizing sites to prevent inter-protein charge recombination or generation of reactive oxygen species, and *in vivo* self-assembly. A solution to these many challenges will require iterative cycles of design, characterization, and analysis to bring us closer to goal of enhanced solar energy conversion through protein design.

## Discussion

We have demonstrated a method of designing helical bundle proteins based on human intuition and experimental evidence of design rules from native and designed proteins. Our approach is sufficient to achieve high-level functions including multi-cofactor binding and photochemical charge separation. New machine learning methods including AlphaFold (Jumper et al., 2021) and RoseTTAFold (Baek et al., 2021) are likely to increase the robustness of this design process, but a firm intuitive foundation will facilitate the effective use of these novel computational tools. We have shown that high-precision rational design of complex four-helix bundles is feasible for human designers without the aid of computational methods.

The RC maquette is a useful testbed to explore photochemical charge separation. Limited promiscuity in cofactor binding allows assembly with different electron donors, pigments, and acceptors that span a range of redox potentials. Crystal structures show that the hydrogen bonding environment involving a redox-active tyrosine can be modified to examine PSII-like tyrosine oxidation. The modular design is amenable to the future reengineering of electron-transfer distances. These attributes enable the same protein scaffold to be used for investigation of electron-transport chains that have many different properties.

The successful modular design and characterization of an RC maquette sets the stage for future functional improvements. We have shown that in principle, a designed RC with a cofactor pentad could trap a charge-separated state with enough energy for water splitting in a single photosystem and with a thermodynamic efficiency surpassing that of natural photosystems. A new RC design with binding sites for five redox centers will need to balance flexibility in cofactor binding pockets with enough apo-state stability to minimize the entropic cost of transitioning to the holo-state. Continued use of the maquette approach to iteratively improve RC maquette thermodynamic and quantum efficiencies, fine tune redox potentials, and introduce catalytic activity may one day lead to new photosynthetic pathways for efficient solar fuel production.

## Data Availability

The datasets presented in this study can be found in online repositories. The names of the repository/repositories and accession number(s) can be found in the article/Supplementary Material.
