# Supplementary material for: Rational design of photosynthetic reaction center protein maquettes
Source: Front Mol Biosci. 2022 Sep 21;9:997295. doi: 10.3389/fmolb.2022.997295 (PMC9532970; doi:10.3389/fmolb.2022.997295)
Supplement: Supplementary file 1 [file DataSheet1.docx]

Supplementary Material

| **Supplementary Table S1: Sequences of designed proteins** | |
| --- | --- |
| RC maquette variant and description | Amino acid sequence |
| Reaction Center (RC) maquette  (original design) | GSPELRQEHQQLAQEFQQLLQEIQQLGRELLKGELQGIKQLREASEKARNPEKKSVLQKILEDEEKHIELLETLQQTGQEAQQLLQELQQTGQELWQLGGSGGPELRQKHQQLAQKIQQLLQKHQQLGAKILEDEEKHIELLETILGGSGGDELRELLKGELQGIKQYRELQQLGQKAQQLVQKLQQTGQKLWQLG |
| RC maquette L71H mutant  (H71-Y168 hydrogen-bonded pair) | GSPELRQEHQQLAQEFQQLLQEIQQLGRELLKGELQGIKQLREASEKARNPEKKSVLQKILEDEEKHIELHETLQQTGQEAQQLLQELQQTGQELWQLGGSGGPELRQKHQQLAQKIQQLLQKHQQLGAKILEDEEKHIELLETILGGSGGDELRELLKGELQGIKQYRELQQLGQKAQQLVQKLQQTGQKLWQLG |
| Di-Heme (DH) maquette  (porphyrin-binding modules only) | GGSPELRQEHQQLAQEFQQLLQEHQQLARELGGSGGDPAEELQQTGQEAQQLLQELQQTGQELWQLGGSGGPELRQKHQQLAQKIQQLLQKHQQLARKLGGSGGDPAEKLQQTGQKAQQLVQKLQQTGQKLWQLG |
| Each protein was expressed with an N-terminal His_6_-tag and TEV protease cleavage sequence of MGKGGHHHHHHGGDGENLYFQ to aid purification. This N-terminal sequence was removed prior to experimentation by TEV protease digestion. | |

| **Supplementary Table S2: Crystallization conditions** | | | | |
| --- | --- | --- | --- | --- |
| crystal structure | protein stock concentration | additives in stock solution | well solution | cryoprotectant* |
| RC maquette with Cd(II)  (no porphyrins)  (PDB ID: 8D9O) | 12.6 mg/mL (560 µM) | 1.5 mM ZnCl_2_ | 24% w/v polyethylene glycol 1500,  100 mM CdCl_2_, 100 mM Na acetate,  pH 4.8 | 2-methyl-2,4-pentanediol |
| RC maquette with Mn(II) and heme B  (PDB ID: 8D9P) | 12.2 mg/mL (540 µM) | 540 µM Heme B,  1 mM MnCl_2_ | 1.2 M Li_2_SO_4_,  0.5 M (NH_4_)_2_SO_4_, 150 mM Na citrate,  pH 5.9 | 28% v/v glycerol,  1.25 M Li_2_SO_4_,  0.5 M (NH_4_)_2_SO_4_,  100 mM Na citrate,  pH 5.85 |
| * Crystals were dipped in the indicated cryoprotectant immediately prior to flash freezing in a nitrogen gas stream at 100 K. | | | | |

| **Supplementary Table S3: Data collection and refinement statistics for X-ray crystal structures** | | |
| --- | --- | --- |
| Protein | RC maquette with Cd(II) (no porphyrins) | RC maquette with Mn(II) and heme B |
| PDB ID | 8D9O | 8D9P |
| **Data Collection** | | |
| Space group | C 1 2 1 | P 4_1_ 2_1_ 2 |
| X-ray source | Rotating Anode Cu K_α_ | Rotating Anode Cu K_α_ |
| Wavelength (Å) | 1.54178 | 1.54178 |
| Unit cell dimensions: |  |  |
| a (Å) | 89.692 | 44.968 |
| b (Å) | 23.693 | 44.968 |
| c (Å) | 72.756 | 238.29 |
| α (°) | 90 | 90 |
| β (°) | 99.93 | 90 |
| γ (°) | 90 | 90 |
| Resolution (Å) | 18.46 – 1.78 (1.829 – 1.783)* | 23.25 – 1.90 (1.95 – 1.90) |
| R_merge_ (%) | 4.7 (17.5) | 8.9 (105.3) |
| I/σ(I) | 17.12 (3.79) | 22.22 (1.66) |
| Completeness (%) | 95.1 (68.9) | 98.4 (86.6) |
| Redundancy | 3.4 (1.7) | 12.6 (5.2) |
| **Refinement** | | |
| Resolution (Å) | 18.46 – 1.78 (1.92 – 1.78) | 23.25 – 1.90 (1.95 – 1.90) |
| Unique reflections | 14,127 (2,331) | 19,019 (1190) |
| Free R value test set selection | Random | Random |
| Free R value test set size (%) | 5.11 | 5.05 |
| R_work_/R_free_ | 0.182/0.208 | 0.224/0.253 |
| Wilson B factor (Å^2^) | 17.7 | 28.2 |
| Mean B value (overall Å^2^) | 25.34 | 37.956 |
| No. of non-H atoms: |  |  |
| All | 1678 | 1709 |
| Protein | 1572 | 1581 |
| Ligand | 11 | 46 |
| Solvent | 95 | 82 |
| Bond RMSD (Å) | 0.014 | 0.009 |
| Angle RMSD (°) | 1.784 | 1.234 |
| Ramachandran favored (%) | 97.4 | 97.9 |
| Ramachandran outliers (%) | 0.0 | 1.0 |
| Rotamer outliers (%) | 0.6 | 0.6 |
| * Values in parentheses represent statistics from the highest resolution shell. | | |

| **Supplementary Table S4: Affinities of various Zn tetrapyrrole pigments for RC maquette-heme B complex** | | |
| --- | --- | --- |
| Pigment | Structure | Dissociation constant (K_D_) |
| Zn 5-phenyl 15-(*p*-carboxyphenyl) porphyrin (ZnPCP)* |  | < 10 nM |
| Zn-SE370† |  | 30 nM |
| Zn Newkome porphyrin* |  | 70 nM |
| Zn-SE375† |  | 70 nM |
| Zn protoporphyrin IX (ZnPPIX) |  | 2 µM |
| Zn meso-tetra(4-carboxyphenyl) prophyrin |  | 5 µM |
| Zn mesoporphyrin IX (ZnMPIX) |  | no binding detected |
| Zn chlorin e_6_ (ZnCe_6_) |  | no binding detected |
| Zn meso-di(4-pyridyl)-diphenyl porphyrin |  | no binding detected |
| * Synthesized by Tatiana Esipova and Sergei Vinogradov group (Kodali et al., 2017).  † Synthesized by Jonathan Lindsey group (Aravindu et al., 2013) (Kodali et al., 2017).  All high-affinity tetrapyrroles bind with stoichiometry of 1.0 to RC maquette with heme B present. | | |

| **Supplementary Table S5: Parameters used for calculation of electron tunneling rates, quantum yields, and thermodynamic efficiencies** | | |
| --- | --- | --- |
| Protein | RC maquette tetrad characterized in this manuscript and in (Ennist et al., 2022) | Hypothetical pentad design |
| Electron Donors | - D_2_ (diiron center) - D_1_ (tyrosinate) | - D_2_ (Mn_4_Ca oxygen-evolving cluster) - D_1_ (tyrosine) |
| Pigment | - P (Zn porphyrin) | - P (Zn porphyrin) |
| Electron Acceptors | - A (heme B) | - A_1_ (tetrapyrrole) - A_2_ (iron sulfur cluster) |
| *Inter-cofactor edge-to-edge distances:* | | |
| D_2_-D_1_ | **3.9 Å*** | 3.9 Å |
| D_1_-P | **4.9 Å** | 4.9 Å |
| P-A_1_ | **13.1 Å** | 5.7 Å |
| A_1_-A_2_ | — | 4.0 Å |
| *Pigment excited state behavior:* | | |
| Singlet (^1^P*) energy | **2.06 eV** | 2.06 eV |
| Triplet (^3^P*) energy | **1.6 eV** | 1.6 eV |
| Intersystem crossing lifetime | 2.9 ns^§^ | 2.9 ns |
| Internal conversion lifetime | 21 ns^§^ | 21 ns |
| Fluorescence lifetime | 80 ns^§^ | 80 ns |
| Phosphorescence lifetime | **4 ms** | 4 ms |
| *Reduction potentials of redox couples (*vs*. SHE):* | | |
| D_2_/D_2_^+^ | ~ +0.5 V | +1.1 V |
| D_1_/D_1_^+^ | ~ +0.72 V | +1.2 V |
| P/P^•+^ | **+0.91 V** | +1.26 V |
| ^1^P*/P^•+^ | **-1.15 V** | -0.80 V |
| ^3^P*/ P^•+^ | **-0.69 V** | -0.34 V |
| A_1_/A_1_^-^ | **-0.19 V** | -0.6 V |
| A_2_/A_2_^-^ | N/A | -0.5 V |
| Reorganization energy (λ) | 0.8 eV | 0.8 eV^\|\|^ |
| Thermodynamic yield | ~33% | 78% |
| Quantum yield 1 ms after laser flash | **6.4%**^†^ | 99.5%^‡^ |
| Lifetime of charge separated state | **250 ms** | 1.9 s |
| * Bold numbers are experimentally measured values (Ennist et al., 2022).  ^§^ Pigment excited state decay lifetimes are based upon typical values of Zn porphyrins reported in (Magdaong et al., 2020).  ^†^ The measured quantum yield of the RC maquette tetrad is the yield of the charge separated state compared to the starting population of ^3^P* excited triplet state (Ennist et al., 2022).  ^‡^ The calculated quantum yield of the pentad design is the yield of the charge separated state compared to the starting population of ^1^P* excited singlet state.  ^\|\|^ Reorganization energy was set to 0.2 eV for lifetimes shorter than the Debye time of 20 ps. | | |

| 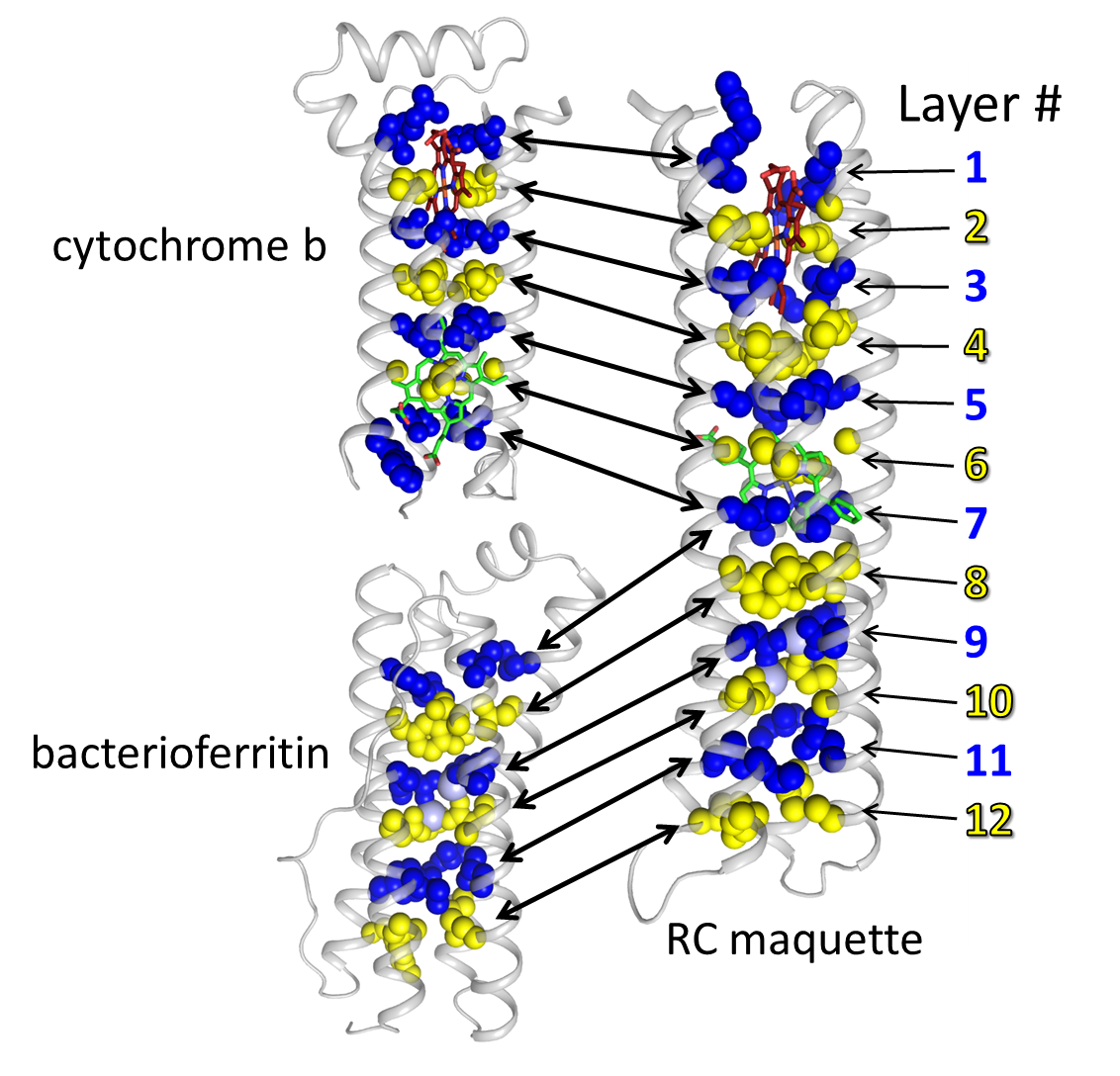 |
| --- |
| **Supplementary Figure S1: Layering in the hydrophobic cores of native and designed proteins.** Heptad positions *a* and *d* are shown as blue or yellow spheres to indicate layers of the hydrophobic core. Each layer contains two *a-* and two *d*-position residues. In the top left is cyt. *b* from PDB ID: 1EZV (Hunte et al., 2000). In the bottom left is bacterioferritin from PDB ID: 1BCF (Frolow et al., 1994). On the right is an RC maquette model based on PDB IDs 5VJS and 5VJU (Ennist et al., 2022) with layers numbered as in Figure 2 of the main text. Arrows between RC maquette design and native crystal structures indicate analogous *a*/*d* layers with similar functional roles. |

| **A**  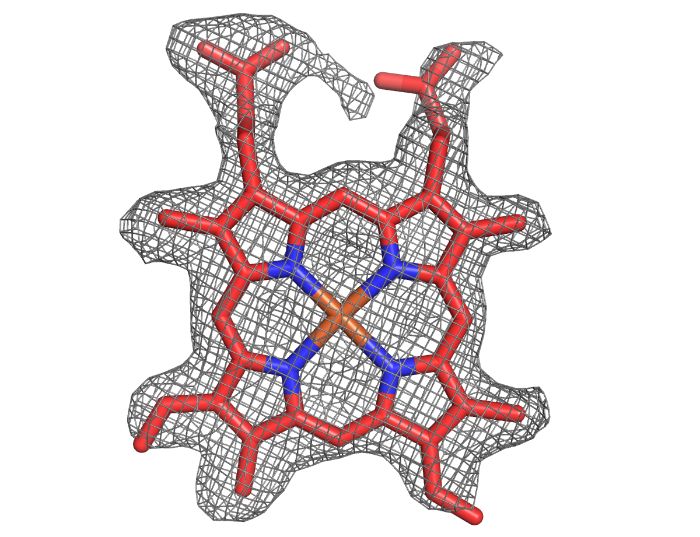 | **B**  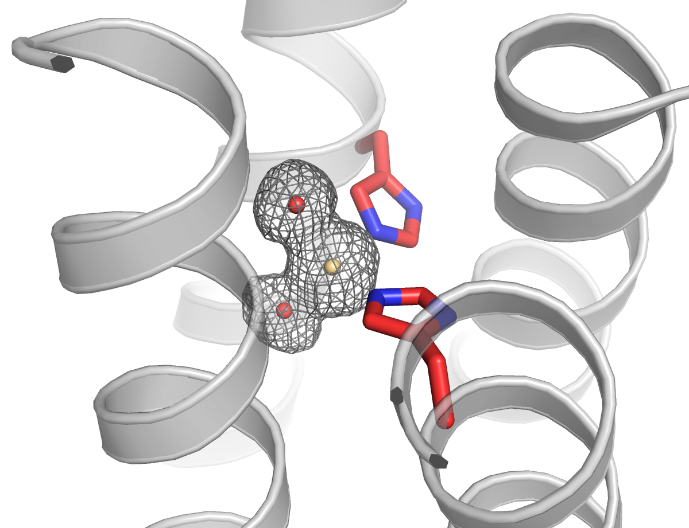 |
| --- | --- |
| **C**  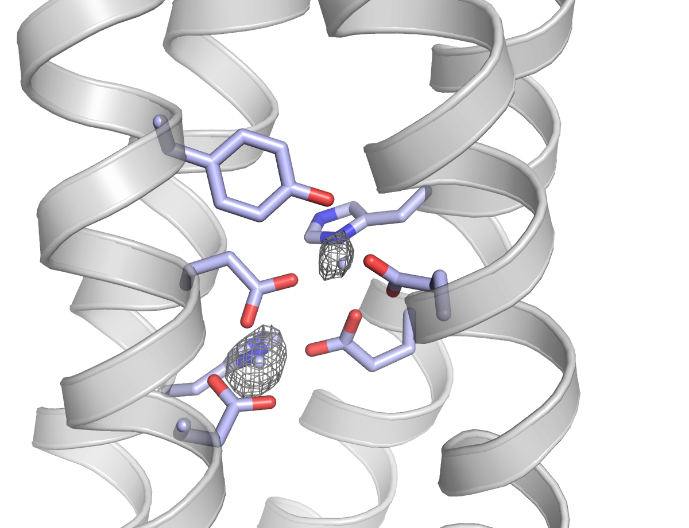 | **D**  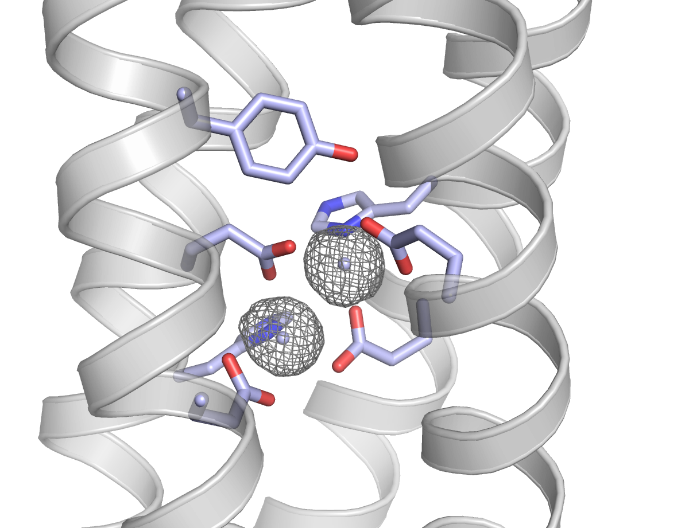 |
| **Supplementary Figure S2: Crystal structure omit maps of cofactor binding sites**. Polder maps are omit maps that exclude bulk solvent around the omitted region. Here, Polder maps (grey mesh) generated in Phenix are used to verify the presence of ligands (Liebschner et al., 2017) (Adams et al., 2010) (Liebschner et al., 2019). All maps are contoured at 3.0 σ. **(A)** Polder map of the A-site heme B molecule (PDB ID: 8D9P). **(B)** Polder map of the A-site Cd(II) ion (tan sphere) and coordinating water molecules (red spheres) (PDB ID: 8D9O). **(C)** Polder map of D-site Mn(II) ions (blue spheres) (PDB ID: 8D9P). The decreased scattering of the Mn(II) ion nearest the Tyr is attributed to its incomplete occupancy of 0.42 in this crystal structure. (**D)** Polder map of D-site Cd(II) ions (blue spheres) (PDB ID: 8D9O). | |

**Supplemental References**

Adams, P.D., Afonine, P.V., Bunkoczi, G., Chen, V.B., Davis, I.W., Echols, N., et al. (2010). PHENIX: A comprehensive Python-based system for macromolecular structure solution. *Acta Crystallogr D Biol Crystallogr* 66 (2)**,** 213-221. doi: 10.1107/S0907444909052925

Aravindu, K., Mass, O., Vairaprakash, P., Springer, J.W., Yang, E., Niedzwiedzki, D.M., et al. (2013). Amphiphilic chlorins and bacteriochlorins in micellar environments. Molecular design, de novo synthesis, and photophysical properties. *Chemical Science* 4**,** 3459-3477. doi: 10.1039/c3sc51335a

Ennist, N.M., Zhao, Z., Stayrook, S.E., Discher, B.M., Dutton, P.L., and Moser, C.C. (2022). De novo protein design of photochemical reaction centers. *Nat Commun* 13(1)**,** 4937. doi: 10.1038/s41467-022-32710-5

Frolow, F., Kalb, A.J., and Yariv, J. (1994). Structure of a unique twofold symmetric haem-binding site. *Nat Struct Biol* 1(7)**,** 453-460.

Hunte, C., Koepke, J., Lange, C., Rossmanith, T., and Michel, H. (2000). Structure at 2.3 A resolution of the cytochrome bc(1) complex from the yeast Saccharomyces cerevisiae co-crystallized with an antibody Fv fragment. *Structure* 8 (6), 669–684. doi:10.1016/s0969-2126(00)00152-0

Kodali, G., Mancini, J.A., Solomon, L.A., Episova, T.V., Roach, N., Hobbs, C.J., et al. (2017). Design and engineering of water-soluble light-harvesting protein maquettes. *Chemical Science* 8(1)**,** 316-324. doi: 10.1039/c6sc02417c

Liebschner, D., Afonine, P.V., Baker, M.L., Bunkoczi, G., Chen, V.B., Croll, T.I., et al. (2019). Macromolecular structure determination using X-rays, neutrons and electrons: Recent developments in Phenix. *Acta Crystallogr D Struct Biol* 75 (10)**,** 861-877. doi: 10.1107/S2059798319011471

Liebschner, D., Afonine, P.V., Moriarty, N.W., Poon, B.K., Sobolev, O.V., Terwilliger, T.C., et al. (2017). Polder maps: improving OMIT maps by excluding bulk solvent. *Acta Crystallogr D Struct Biol* 73 (2)**,** 148-157. doi: 10.1107/S2059798316018210

Magdaong, N.C.M., Taniguchi, M., Diers, J.R., Niedzwiedzki, D.M., Kirmaier, C., Lindsey, J.S., et al. (2020). Photophysical properties and electronic structure of zinc(II) porphyrins bearing 0-4 meso-phenyl substituents: Zinc porphine to zinc tetraphenylporphyrin (ZnTPP). *J Phys Chem A* 124(38)**,** 7776-7794. doi: 10.1021/acs.jpca.0c06841
